# Glial Chloride Homeostasis Under Transient Ischemic Stress

**DOI:** 10.3389/fncel.2021.735300

**Published:** 2021-09-16

**Authors:** Miriam Engels, Manu Kalia, Sarah Rahmati, Laura Petersilie, Peter Kovermann, Michel J. A. M. van Putten, Christine R. Rose, Hil G. E. Meijer, Thomas Gensch, Christoph Fahlke

**Affiliations:** ^1^Institute of Biological Information Processing, Molekular-und Zellphysiologie (IBI-1), Forschungszentrum Jülich, Jülich, Germany; ^2^Applied Analysis, Department of Applied Mathematics, University of Twente, Enschede, Netherlands; ^3^Institute of Neurobiology, Heinrich Heine University Düsseldorf, Düsseldorf, Germany; ^4^Department of Clinical Neurophysiology, University of Twente, Enschede, Netherlands

**Keywords:** intracellular chloride concentrations, chemical stress mimicking ischemia, fluorescence lifetime imaging microscopy, excitatory amino acid transporters, Na-K-2Cl cotransporter, K-Cl cotransporters

## Abstract

High water permeabilities permit rapid adjustments of glial volume upon changes in external and internal osmolarity, and pathologically altered intracellular chloride concentrations ([Cl^–^]_int_) and glial cell swelling are often assumed to represent early events in ischemia, infections, or traumatic brain injury. Experimental data for glial [Cl^–^]_int_ are lacking for most brain regions, under normal as well as under pathological conditions. We measured [Cl^–^]_int_ in hippocampal and neocortical astrocytes and in hippocampal radial glia-like (RGL) cells in acute murine brain slices using fluorescence lifetime imaging microscopy with the chloride-sensitive dye MQAE at room temperature. We observed substantial heterogeneity in baseline [Cl^–^]_int_, ranging from 14.0 ± 2.0 mM in neocortical astrocytes to 28.4 ± 3.0 mM in dentate gyrus astrocytes. Chloride accumulation by the Na^+^-K^+^-2Cl^–^ cotransporter (NKCC1) and chloride outward transport (efflux) through K^+^-Cl^–^ cotransporters (KCC1 and KCC3) or excitatory amino acid transporter (EAAT) anion channels control [Cl^–^]_int_ to variable extent in distinct brain regions. In hippocampal astrocytes, blocking NKCC1 decreased [Cl^–^]_int_, whereas KCC or EAAT anion channel inhibition had little effect. In contrast, neocortical astrocytic or RGL [Cl^–^]_int_ was very sensitive to block of chloride outward transport, but not to NKCC1 inhibition. Mathematical modeling demonstrated that higher numbers of NKCC1 and KCC transporters can account for lower [Cl^–^]_int_ in neocortical than in hippocampal astrocytes. Energy depletion mimicking ischemia for up to 10 min did not result in pronounced changes in [Cl^–^]_int_ in any of the tested glial cell types. However, [Cl^–^]_int_ changes occurred under ischemic conditions after blocking selected anion transporters. We conclude that stimulated chloride accumulation and chloride efflux compensate for each other and prevent glial swelling under transient energy deprivation.

## Introduction

Glial cells fulfill a variety of important functions in the mammalian central nervous system. First, they supply nutrient and signaling molecules to neurons and regulate extracellular K^+^ concentrations (Deitmer and Rose, 2010). Second, glial secondary active transport systems control resting synaptic neurotransmitter concentrations to optimize the spatiotemporal resolution of synaptic transmission. Glial cells have higher water permeability than neurons, making them prone to faster changes in cell volume caused by physiological variations in external and internal osmolarity (Andrew et al., 2007; MacAulay and Zeuthen, 2010; Nagelhus and Ottersen, 2013; Papadopoulos and Verkman, 2013). Since changes in osmotically active solute concentrations are intimately associated with glial key functions, mechanisms for volume regulation are especially important for this class of cells. Various pathological conditions (such as epilepsy, hepatic failure, hyponatremia, stroke, and traumatic brain and spinal cord injuries) can result in dysregulation of astrocytic cell volume; astrocyte swelling may induce cytotoxic brain edema (Kimelberg, 2005; Stokum et al., 2016; Wilson and Mongin, 2018). By reducing the extracellular volume, glial swelling has the potential to modify metabolite and neurotransmitter diffusion in the extracellular space. Moreover, increased intracranial pressure can result in life-threatening conditions such as tissue damage and reduced blood flow.

The intracellular chloride concentration ([Cl^–^]_int_) represents a main determinant of volume regulation (Dijkstra et al., 2016). Intracellular accumulation of NaCl and KCl drives the inward movement of water during cell swelling (Lang et al., 1998; Mongin and Orlov, 2001; Pasantes-Morales, 2016). Volume recovery after cell swelling is based on water efflux driven by the synchronized outward movement of K^+^ and Cl^–^ or HCO_3_^–^ without affecting the transmembrane voltage (Kahle et al., 2015; Mongin, 2016; Delpire and Gagnon, 2018; Wilson and Mongin, 2018; Toft-Bertelsen et al., 2021). However, the mechanisms underlying glial chloride homeostasis remain insufficiently understood.

Here we used fluorescence lifetime imaging microscopy (FLIM) with the chloride-sensitive dye MQAE (Kaneko et al., 2004; Kovalchuk and Garaschuk, 2012; Gensch et al., 2015; Untiet et al., 2017) to study [Cl^–^]_int_ in glial cells under both control conditions and conditions that mimic ischemic energy restriction. We determined the resting [Cl^–^]_int_ in four types of glial cells [hippocampal astrocytes in the dentate gyrus (DG) and cornu ammonis region 1 (CA1), hippocampal radial glia-like (RGL) cells, and neocortical astrocytes] in acute brain slices and observed marked regional heterogeneity in glial ion concentration. Using specific blockers, we identified the key chloride transport proteins that determine glial chloride homeostasis and assessed their contribution to [Cl^–^]_int_ and cell volume in the tested brain regions. Although chloride transport depends on processes that are affected during ischemia, we observed only slight absolute changes in [Cl^–^]_int_ upon transient chemical ischemia. To explain our results, we used a mathematical model – recently established to describe the ion dynamics at the tripartite synapses (Kalia et al., 2021) – to define mechanisms of chloride homeostasis under normal as well as under energy deprivation. This model is based on ion concentration measurements at room temperature, and all our experiments were performed at this temperature.

## Materials and Methods

### Animals

Animals were housed under standard conditions in the animal facility of Forschungszentrum Jülich (SV129) or Heinrich Heine University Düsseldorf (Balb/C) according to institutional guidelines under a 12-h light/dark cycle and in small groups with food and water provided *ad libitum*.

### Fluorescence Lifetime Imaging Microscopy (FLIM)

After decapitation under isoflurane anesthesia brains were rapidly removed and placed in oxygenated, ice-cold preparation solution containing (in mM) 125 NaCl, 2.5 KCl, 1.25 NaH_2_PO_4_, 26 NaHCO_3_, 0.5 CaCl_2_, 5 MgCl_2_, and 25 glucose. Sagittal hippocampal or coronal cortical slices (250 μm thickness) were cut with a microtome (Microm HM650V, Thermo Scientific, Walldorf, Germany; frequency 60 Hz, amplitude 1 mm, drive 10) and transferred to a gauze slice holder in oxygenated Ringer’s solution at 37°C (carbogen; 95% O_2_/5% CO_2_, Untiet et al., 2017). Glial cells were stained with sulforhodamine 101 (SR101, Sigma-Aldrich, St. Louis, MO, United States) in a preparation Ringer’s solution that contained 2 μM SR101 for 20 min at 37°C (Kafitz et al., 2008), followed by a 10 min incubation in standard oxygenated Ringer’s solution with (in mM) 125 NaCl, 2.5 KCl, 1.25 NaH_2_PO_4_, 26 NaHCO_3_, 2 CaCl_2_, 1 MgCl_2_, and 20 glucose, at 37°C. The stained acute tissue slices were kept at room temperature (22–24°C) for at least 30 min before use. Slices were constantly perfused with oxygenated standard Ringer’s solution. All experiments were performed within 5–8 h of brain removal.

Brain slices were incubated in oxygenated standard Ringer’s solution containing 3.5 mM 1-(ethoxycarbonylmethyl)-6-methoxyquinolinium bromide (MQAE, Sigma-Aldrich, Merck, Darmstadt, Germany) (Verkman, 1990) for 30–40 min at room temperature and then transferred to an imaging chamber attached to an upright fluorescence microscope. Experiments were performed using two different imaging systems, since we acquired a new two-photon excitation fluorescence microscope with FLIM modality during the course of this study. Roughly half of the experiments utilized an A1 MP microscope (Nikon, Amsterdam, Netherlands) equipped with a 25× water immersion objective [NA1.1; working distance (WD) 2 mm; XYZ, Nikon] and a mode-locked Titan-Sapphire laser (Mai Tai DeepSee, Newport Spectra Physics; Irvine, CA, United States; output power 2.3 W at 750 nm); the other half of the experiments were performed on a LSM880 microscope (Zeiss, Jena, Germany) equipped with a 20× water immersion objective (NA 1.0, WD 2.1 mm; XYZ, Zeiss) linked to a tunable laser (InSight X3, Newport Spectra Physics, Darmstadt, output power 1.9 W at 750 nm). Two-photon excitation (λ_exc_ = 750 nm) was carried out at 80 MHz with either 100 fs light pulses or 120 fs light pulses, with identical results. Mean fluorescence lifetimes were measured using multidimensional time-correlated single-photon counting (TCSPC) in a volume of 0.08 μm^3^ per individual pixel, resulting in a three-dimensional resolution of approximately 1.3 μm in the *z*-axis and 0.35 μm in the *x*- and *y*-axes (Zipfel et al., 2003).

Six to eight images were taken for each measurement, starting at a depth of about 30 μm from the upper cutting edge and reaching not further than approximately 100 μm into the slice. For all brain regions studied, we only included glial cells in the analysis that were clearly stained by SR101, sufficiently loaded with MQAE, and showed region-specific characteristic morphology. Following two-photon (Nikon A1 MP microscope: 900 nm) or one-photon (Zeiss LSM880 confocal microscope: 561 nm) excitation, emitted SR101 fluorescence was filtered using an appropriate bandpass filter (Nikon A1 MP: 595AF60, 565–637 nm; Omega Optical, Brattleboro, VT, United States) or a GaAsP detector (Zeiss, LSM880) with a freely selectable emission detection band (570–700 nm). A single region of interest (ROI) was defined for each glial soma, and the fluorescence lifetime of cells was calculated as the average fluorescence lifetime (τ_ave_) of all pixels in the ROI. Fluorescence was filtered by separating MQAE fluorescence (peak emission: 460 nm) from autofluorescence (bandpass 445bp90: 400–490 nm; Omega Optical, Brattleboro, VT, United States) and recorded with a GaAsP hybrid photodetector (HPM-100-40, Becker & Hickl, Berlin, Germany) with non-descanned detection. TCSPC electronics (SPC-152; Becker & Hickl, Berlin, Germany) and acquisition software were used for FLIM as previously described (Kaneko et al., 2004; Untiet et al., 2017).

**FIGURE 1:**
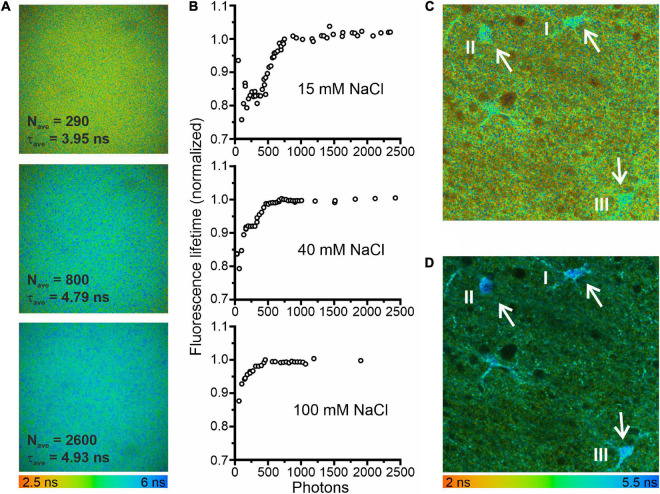
Minimum photon numbers for the determination of fluorescence lifetime by fitting fluorescence decays. **(A)** Fluorescence lifetime images in a droplet of 3 mM MQAE dissolved in a solution containing 15 mM NaCl. The three images were calculated from the same data set using different bin factors to generate single-pixel fluorescence intensity decays (FIDs) with varying photon numbers. **(B)** Fluorescence lifetimes of 3 mM MQAE (15 mM NaCl normalized to the limiting value of 4.95 ns; 40 mM NaCl normalized to the limiting value of 2.2 ns; 100 mM NaCl normalized to the limiting value of 1.15 ns) plotted as a function of photon numbers per FID. Photon numbers per FID varied by the excitation power, acquisition time, or bin factor. **(C)** Fluorescence lifetime image of a hippocampal brain slice (CA1 region). The average photon numbers for single-pixel FIDs in the three glia cells visible in the center of the image were 296 (I), 269 (II), and 373 (III), resulting in reduced fluorescence lifetimes: 4.22 ns (I), 4.24 ns (II), and 4.15 ns (III). **(D)** A second fluorescence lifetime image of the same brain slice area, measured with a longer acquisition time and otherwise identical measurement conditions. Consequently, the respective FID photon numbers were significantly higher 4817 (I), 3941 (II), and 6240 (III), resulting in expected fluorescence lifetimes: 4.88 ns (I), 4.96 ns (II), and 4.73 ns (III).

Fluorescence lifetime images were generated using SPCImage 6.0–8.3 (Becker & Hickl, Berlin, Germany). We usually summed fluorescence intensity decays (FIDs) over nine pixels and assigned the resulting value to the central pixel (bin factor 1). In a few cases, in which the number of photons per pixel was below the critical value of 2000 counts (Figure 1), we used a bin factor of 2 (i.e., we summed photon distributions over the central and the surrounding 24 pixels). Fluorescence decays were fitted with bi-exponential functions (Kaneko et al., 2004; Gilbert et al., 2007; Funk et al., 2008; Untiet et al., 2017), and the average fluorescence lifetime (τ_ave_)


$$\tau_{ave}\;=\;\frac{a_{1}\cdot \tau_{1}+a_{2}\cdot \tau_{2}}{a_{1}+a_{2}}$$

was used to calculate [Cl^–^]_int_ values (τ_x_ = lifetime of the exponential component; a_x_ = respective amplitude).

**FIGURE 2:**
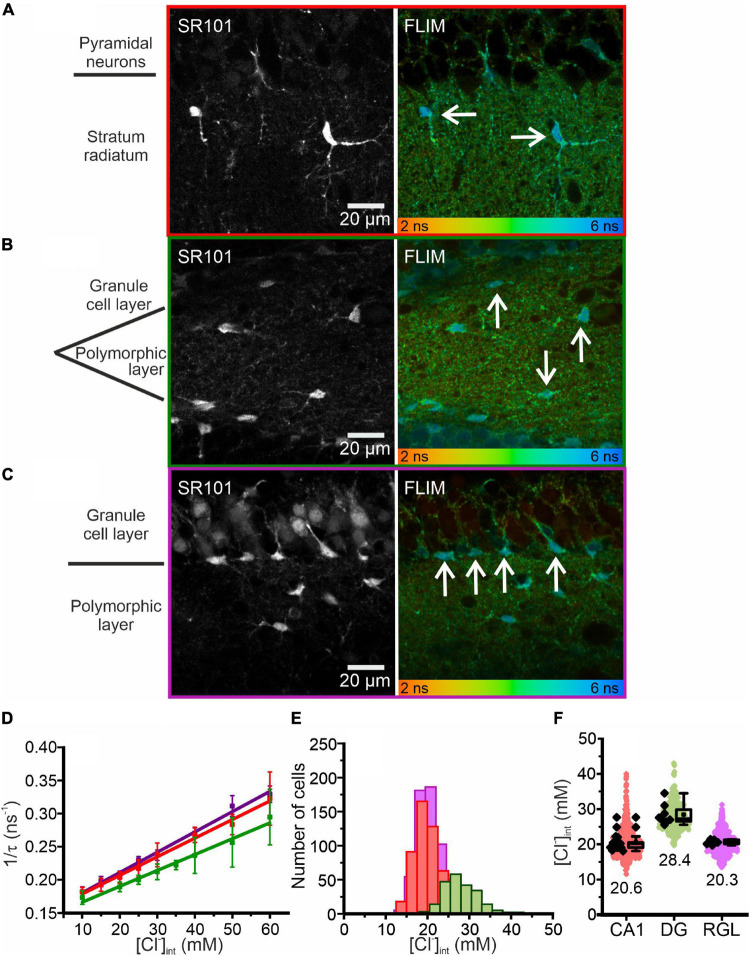
Chloride imaging in acute hippocampal slices. **(A–C)** SR101 stained astrocytes and representative fluorescence lifetime images (FLIM) of the CA1 **(A)** and DG **(B)** regions and RGL cells **(C)**, with glial cells marked by arrows. Average fluorescence lifetimes (in ns) are color-coded. **(D)** Stern–Volmer plot of the chloride dependence of the MQAE fluorescence lifetime in hippocampal glia. Data points represent the inverse average fluorescence lifetime at different chloride concentrations, error bars indicate standard deviation, and solid lines represent a linear fit (10 – 60 mM; *N* = 3 animals/chloride concentration, >10 cells/mouse/chloride concentration, mean ± SD). **(E,F)** Physiological [Cl^−^]_int_ of all three glial cell types shown as a histogram **(E)** or box plot **(F)** (red – CA1, green – DG, purple – RGL). In the box plot (mean ± 1.5 IQR), black points are the mean [Cl^−^]_int_ from individual animals and colored points are the mean [Cl^−^]_int_ from individual cells. Abbreviations: CA1, cornu ammonis region 1; DG, dentate gyrus; RGL, radial glial-like cells.

Fitting the sum of exponentials to fluorescence decay curves from TCSPC experiments can result in systematic errors for low photon counts (Maus et al., 2001). To define conditions that circumvent this limitation of FLIM-based concentration measurements, we studied the consequences of low photon numbers with the two-photon fluorescence microscopes with FLIM modality used for measuring glial [Cl^–^]_int_ (Figure 1). Using of droplets of aqueous MQAE solutions with [Cl^–^] of 15, 40, or 100 mM, we found that at high photon numbers (>1000 photons) the determined fluorescence lifetime is identical to the fluorescence lifetime determined independently with a cuvette-based fluorescence lifetime spectrophotometer (TimeHarp 100, PicoQuant, Berlin, Germany) and is not modified by further increasing the photon count. We then modified the photon number of FIDs by changing the applied binning options. Smaller binning reduced the photon numbers per decay and significantly decreased fluorescence lifetimes (Figure 1A). We combined binning with variation of the excitation power and found that – for less than 1000 photons per FID – the estimated fluorescence lifetime deviates by up to 25% from the correct value (Figure 1B). In similar experiments with two other chloride concentrations (40 mM NaCl and 100 mM NaCl) and shorter fluorescence lifetimes similar, but less pronounced effects of low photon numbers were observed (Figure 1B). A similar effect of low photon counts was observed in experiments with glial cells in the CA1 region of an acute hippocampal slice [Figure 1C (three cells), I: 4.22 ns (296 photons per FID); II: 4.24 ns (269 photons per FID); III: 4.15 ns (473 photons per FID)]. These lifetimes predict incorrectly high chloride concentrations (I: 30.8 mM; II: 30.3 mM; III: 32.1 mM). When the same area was measured with a longer acquisition time, photon numbers per FID were well above 1000 and we obtained the expected fluorescence lifetimes [Figure 1D, I: 4.88 ns (4817 photons per FID); II: 4.96 ns (3941 photons per FID); III: 4.73 ns (6240 photons per FID)], as well as chloride concentrations (I: 19.3 mM; II: 18.1 mM; III: 21.6 mM) near to the mean of [Cl^–^]_int_ estimated for hippocampal (CA1) glia cells (Figure 2F). Therefore, we chose to use a minimum number of 2000 photons per FID in all of our experiments.

**FIGURE 3:**
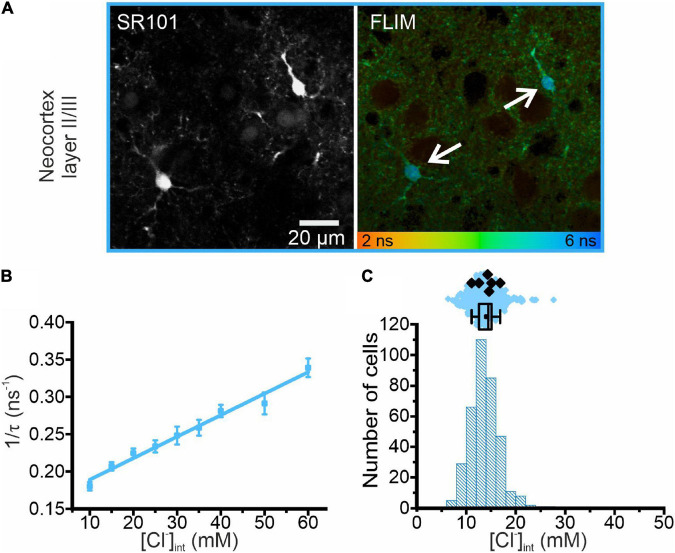
Chloride imaging of neocortical layer II/III astrocytes. **(A)** SR101 stained astrocytes and representative fluorescence lifetime image (FLIM) of neocortical astrocytes (marked by arrows). Average fluorescence lifetimes (in ns) are color-coded. **(B)** Stern–Volmer plot of the chloride dependence of the MQAE fluorescence lifetime in neocortical astrocytes. Data points represent the inverse average fluorescence lifetime measured at different chloride concentrations, error bars indicate the standard deviation, and solid lines represent a linear fit (10 – 60 mM; *N* = 3 animals/chloride concentration, >10 cells/mouse/chloride concentration, mean ± SD). **(C)** Physiological [Cl^−^]_int_ in neocortical astrocytes, shown as a histogram and box plot. In the box plot (mean ± 1.5 IQR), black points are the mean [Cl^−^]_int_ from individual animals and colored points are the mean [Cl^−^]_int_ from individual cells.

For calibration (Figures 2D, 3B) acute tissue slices were incubated in HEPES-buffered solutions containing (in mM) 140 K^+^, 10 Na^+^, 10 HEPES, 10–60 Cl^–^, 80–130 gluconate, adjusted to 310 mOsm/L with K-gluconate and to pH 7.4 with KOH, supplemented with 20 μM nigericin (sodium salt; Sigma-Aldrich, Merck, Darmstadt, Germany) and 20 μM tributyltin (chloride salt; Sigma-Aldrich, Merck, Darmstadt, Germany) (Chao et al., 1989; Bevensee et al., 1997; Kaneko et al., 2004; Kovalchuk and Garaschuk, 2012). Since MQAE is quenched much less effectively by HCO_3_^–^ than by Cl^–^ (Kaneko et al., 2004), intracellular bicarbonate is not expected to contribute to the MQAE lifetime and was therefore ignored in calibration experiments.

**FIGURE 4:**
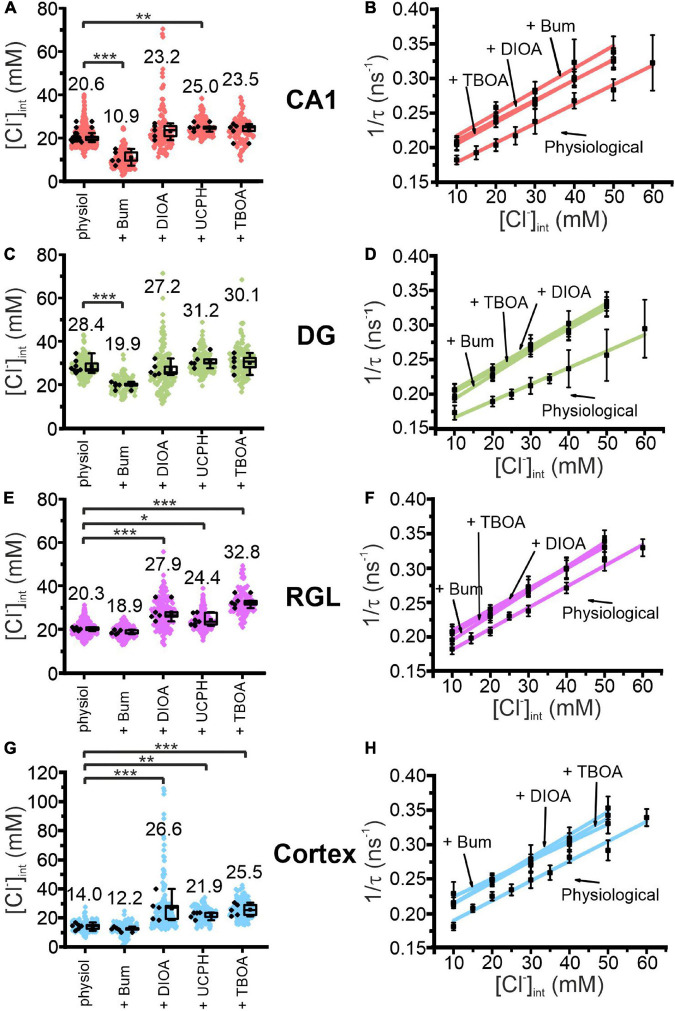
Modification of glial resting [Cl^−^]_int_ and chloride dependence of MQAE fluorescence lifetimes by indicated blocker. [Cl^−^]_int_ of hippocampal astrocytes in the CA1 **(A)** and DG **(C)** regions, RGL cells **(E)**, and neocortical glial cells **(G)** under physiological conditions or after treatment with blocker, as indicated. In the box plot (mean ± 1.5 IQR), black points are the mean [Cl^−^]_int_ from individual animals and colored points are the mean [Cl^−^]_int_ from individual cells. Stern–Volmer plots for glial cells in the CA1 **(B)** and DG **(D)** regions, RGL cells of the hippocampus **(F)**, and neocortical glial cells **(H)**. For each brain region, Stern–Volmer plots were created under physiological conditions and in the presence of bumetanide, *R*-(+)-DIOA, or DL-TBOA to visualize the influence of these blockers on MQAE fluorescence lifetimes. Data points represent the inverse average fluorescence lifetime at different chloride concentrations, error bars indicate the standard deviation, and the solid lines represent a linear fit (10–60 mM; *N* = 3 animals/chloride concentration, >10 cells/mouse/chloride concentration, mean ± SD). *p ≤ 0.05, **p ≤ 0.01, ***p ≤ 0.001, one-way ANOVA with Holm–Sidak *post hoc* test; all *p*-values for this figure are mentioned in the results. Abbreviations: CA1, cornu ammonis region 1; DG, dentate gyrus; RGL, radial glial-like cells; Physiol, physiological; Bum, bumetanide; DIOA, *R*-(+)-DIOA; UCPH, UCPH-101; TBOA, DL-TBOA.

DL-threo-β-benzyloxyaspartic acid (DL-TBOA) (100 μM; Tocris Bioscience, Bristol, United Kingdom), *R*-(+)-butylindazone, (dihydroindenyl)oxy alkanoic acid (*R*-(+)-DIOA, 100 μM, Sigma-Aldrich, Merck, Darmstadt, Germany) or 3-butylamino-4-phenoxy-5-sulfamoyl benzoic acid (bumetanide; 40 μM, Sigma-Aldrich, Merck, Darmstadt, Germany) were added to both the incubation (20 min incubation) and perfusion solutions. In contrast, 2-amino-5,6,7,8-tetrahydro-4-(4-methoxyphenyl)-7-(naphthalen-1-yl)-5-oxo-4*H*-chromene-3-carbonitrile (UCPH-101, Abcam, Cambridge, United Kingdom) inhibits excitatory amino acid transporter 1 (EAAT1/GLAST) with very slow unblocking kinetics (Abrahamsen et al., 2013) and is not washed out within the imaging experiment duration (around 30 min). Hence, in relevant experiments, brain slices were incubated with 20 μM UCPH-101 for 20 min and then perfused with standard Ringer’s solution without UCPH-101 for imaging. MQAE fluorescence is quenched not only collisionally by chloride ions (Verkman, 1990), but also by the chemical blockers used in our experiments. Consequently, to account for changes in fluorescence lifetimes, we performed additional calibrations after 20 min incubation with blockers (Figures 4B,D,F,H). The importance of these additional calibration was proven by differences in [Cl^–^]_int_ calculated using the original calibration (without blocker) compared with corrected values after additional calibration. Re-calibration of MQAE lifetimes with three blockers (bumetanide, *R*-(+)-DIOA, and DL-TBOA) is necessary to accurately assess the roles of specific chloride transporters/channels in setting the [Cl^–^]_int_. Since MQAE self-quenching reduces the fluorescence lifetime (at fixed [Cl^–^]) with increasing [MQAE] (Kaneko et al., 2002; Gensch et al., 2015), we performed calibration experiments using the same MQAE loading protocols as in the actual measurements.

Transient energy restriction in the ischemic penumbra was mimicked by removing glucose from standard Ringer’s solution and adding 5 mM NaN_3_ and 2 mM 2-deoxy-D-glucose (Sigma-Aldrich) (Gerkau et al., 2018). Slices were perfused for 2, 5, or 10 min with the ischemia cocktail, and then continuously perfused with oxygenated standard Ringer’s solution during imaging. FLIM measurements under ischemic conditions were always performed as the first experiments of the day, with two to five slices tested for different ischemic periods (2, 5, or 10 min). Fluorescence lifetimes were determined with scanning times of at least 40 or 80 s. Slices with insufficient MQAE loading or strong slice movement during scanning were not included in the analysis. We approximated glial cell volumes from FLIM images by defining a circular ROI surrounding the glial soma and assuming a spherical shape for the glial cell soma (Supplementary Figures 1–3).

### Cell Volume Measurements Based on Maximum Intensity Projections

P14–P18 mice (both sexes) were killed by CO_2_ anesthesia and decapitation. The brains were removed and placed in oxygenated, ice-cold preparation saline (in mM: 125 NaCl, 2.5 KCl, 0.5 CaCl_2_, 6 MgCl_2_, 1.25 NaH_2_PO_4_, 26 NaHCO_3_, and 20 glucose). Parasagittal brain slices (250 μm) were obtained using a vibratome (Microm HM 650V, Thermo Scientific, Planegg, Germany or 7000smz-2, n.p.i.Tamm, Germany) and incubated for 20 min at 34°C in standard oxygenated Ringer’s solution. During the incubation period, SR101 (0.5–1 μM) was added to selectively stain astrocytes (Kafitz et al., 2008). After staining, brain slices were maintained in standard Ringer’s solution at room temperature until use.

During experiments, brain slices were continuously perfused with standard Ringer’s solution at room temperature. Hypo-osmotic stress was induced by reducing the NaCl concentration of standard Ringer’s solution to 75 mM, thereby decreasing the osmolarity from about 310–220 mOsm/L. Chemical ischemia was induced by perfusing slices with glucose-free standard Ringer’s solution containing 5 mM NaN_3_ and 2 mM 2-deoxy-D-glucose (Gerkau et al., 2018). A motorized confocal laser scanning microscope (Nikon Eclipse C1: Fluor 60×/1.00 W, software EZ-C1 3.91 Nikon Instruments, Düsseldorf, Germany) was used to document SR101 fluorescence. Z-stacks were taken at 0.6 μm increments and maximum intensity projections (MIPs) were calculated in ImageJ. Images were deconvolved using Huygens Professional Software (Scientific Volume Imaging, Hilversum, Netherlands).

### Modeling [Cl^–^]_int_ Under Transient Ischemia

To simulate ischemia-induced changes in [Cl^–^]_int_, we modified a recently developed model (Kalia et al., 2021) that describes the temporal evolution of intra- and extracellular Na^+^, K^+^, Cl^–^, Ca^2+^ and glutamate concentrations in neurons and astrocytes using the following differential equations:


$$\frac{d}{dt}N_{x}^{i}=-\frac{1}{z_{x}F}\sum_{j}I_{j}^{x,i},$$

$$\frac{d}{dt}W_{i}=\lambda_{i}\sum_{x}\left({\left[X\right]}_{i}-{\left[X\right]}_{e}\right),$$

$$\frac{d}{dt}q=\alpha_{q}\left(1-q\right)-\beta_{q}q,$$

with $N_{x}^{i}$ denoting the number of moles of *X* in compartment *i*, *z*_X_ the valence of ions *X*, and *F* Faraday’s constant. The term $I_{j}^{x,i}$ describes currents mediated by the transporter *j* with respect to ion *X* in compartment *i*. The compartments are allowed to swell and shrink in response to osmotic gradients across the membrane. Volumes *W*_i_ of both compartments and molar ionic amounts were used to determine the ion concentrations. The term *q* denotes several voltage-dependent gates that are used to model some of the ion channels.

Currents $I_{j}^{x,i}$*^*i*^* mediated by ion exchangers/cotransporters or channels were modeled as described in Kalia et al. (2021). In the neuron, the following currents were considered:

1. voltage-gated Na^+^, K^+^, Cl^–^ and Ca^2+^ channels generating $I_{G}^{X,n}$;
2. Na^+^/K^+^-ATPases (NKA) generating $I_{NKA}^{{\mathrm{Na}}^{+},n}$ and $I_{NKA}^{K^{+},n}$;
3. K^+^-Cl^–^-cotransporters (KCC) generating $I_{KCC}^{K^{+},n}$ and $I_{KCC}^{{\mathrm{Cl}}^{-},n}$
4. Na^+^/Ca^2+^-exchangers (NCX) generating $I_{NCX}^{{\mathrm{Na}}^{+},n}$ and $I_{NCX}^{{\text{Ca}}^{2+},n}$; and
5. excitatory amino acid transporters (EAAT) generating $I_{EAAT}^{{\mathrm{Na}}^{+},n}$, $I_{EAAT}^{K^{+},n}$, and $I_{EAAT}^{Glu,n}$.

For astrocytes the model includes the following currents:

1. Kir4.1 channel generating $I_{Kir}^{K^{+},a}$;
2. Na^+^/K^+^-ATPase (NKA) generating $I_{NKA}^{{\mathrm{Na}}^{+},a}$ and $I_{NKA}^{K^{+},a}$;
3. Na^+^-K^+^-2Cl^–^-cotransporter (NKCC1) generating $I_{NKCC}^{{\mathrm{Na}}^{+},a}$, $I_{NKCC}^{K^{+},a}$ and $I_{NKCC}^{{\mathrm{Cl}}^{-},a}$;
4. Na^+^/Ca^2+^-exchanger (NCX) generating $I_{NCX}^{{\mathrm{Na}}^{+},a}$ and $I_{NCX}^{{\text{Ca}}^{2+},a}$; and
5. excitatory amino acid transporter (EAAT) generating $I_{EAAT}^{{\mathrm{Na}}^{+},a}$, $I_{EAAT}^{K^{+},a}$ and $I_{EAAT}^{Glu,a}$

Our model differs from the earlier version (Kalia et al., 2021) by the addition of KCC transporters as the Cl^–^ transport system in the astrocytic plasma membrane, in addition to the NKCC1 and Cl^–^ leak channels. Similarly, to the neuronal KCC, we assumed that the transport number *J*_KCC_ changes proportionally to the driving force of this coupled transporter, as follows:


JKCCa=PKCCaRTFln⁡([K+]e[K+]i[Cl-]e[Cl-]i).

This gives rise to the additional currents $I_{KCC}^{K^{+},a}$ and $I_{KCC}^{{\mathrm{Cl}}^{-},a}$ with


$$I_{KCC}^{K^{+},a}=FJ_{KCC}^{a}$$

$$I_{KCC}^{{\mathrm{Cl}}^{-},a}=-FJ_{KCC}^{a}.$$

We did not explicitly model the glial anion conductances generated by EAAT anion channels; these anion efflux pathways are represented as components of the anion leak conductance of the modeled glial cells.

Our model describes the dynamics of molar amounts $N_{X}^{i}$ of the ions Na^+^, K^+^, Cl^–^, Ca^2+^, and glutamate, and compartmental volumes *W*_i_, for *i* = {*n*, *a*}.


$$\frac{d}{dt}N_{Na^{+}}^{n}=\frac{1}{F}\left(I_{G}^{{\mathrm{Na}}^{+},n}+I_{NKA}^{{\mathrm{Na}}^{+},n}+I_{EAAT}^{{\mathrm{Na}}^{+},n}+I_{NCX}^{{\mathrm{Na}}^{+},n}+I_{L}^{{\mathrm{Na}}^{+},n}\right)+\frac{1}{F}I_{stim}\left(t\right),$$

$$\frac{d}{dt}N_{K^{+}}^{n}=\frac{1}{F}\left(I_{G}^{K^{+},n}+I_{NKA}^{K^{+},n}+I_{EAAT}^{K^{+},n}+I_{KCC}^{K^{+},n}+I_{L}^{K^{+},n}\right),$$

$$\frac{d}{dt}N_{{\mathrm{Cl}}^{-}}^{n}=\frac{1}{F}\left(I_{G}^{{\mathrm{Cl}}^{-},n}+I_{KCC}^{{\mathrm{Cl}}^{-},n}+I_{L}^{{\mathrm{Cl}}^{-},n}\right),$$

$$\frac{d}{dt}N_{Ca^{2+}}^{n}=\frac{1}{2F}\left(I_{G}^{Ca^{2+},n}+I_{NCX}^{Ca^{2+},n}+I_{L}^{Ca^{2+},n}\right),$$

$$\frac{d}{dt}N_{Glu}^{n}=\frac{1}{F}\left(I_{EAAT}^{Glu,n}+I_{L}^{Glu,n}\right),$$

$$\frac{d}{dt}N_{Na^{+}}^{a}=\frac{1}{F}\left(I_{NKCC}^{{\mathrm{Na}}^{+},a}+I_{NKA}^{{\mathrm{Na}}^{+},a}+I_{EAAT}^{{\mathrm{Na}}^{+},a}+I_{NCX}^{{\mathrm{Na}}^{+},a}+I_{L}^{{\mathrm{Na}}^{+},a}\right),$$

$$\frac{d}{dt}N_{K}^{a^{+}}=\frac{1}{F}\left(I_{NKCC}^{K^{+},a}+I_{NKA}^{K^{+},a}+I_{EAAT}^{K^{+},a}+I_{Kir}^{K^{+},a}+I_{KCC}^{K^{+},a}+I_{L}^{K^{+},a}\right),$$

$$\frac{d}{dt}N_{{\mathrm{Cl}}^{-}}^{a}=\frac{1}{F}\left(I_{NKCC}^{{\mathrm{Cl}}^{-},a}+I_{KCC}^{{\mathrm{Cl}}^{-},a}+I_{L}^{{\mathrm{Cl}}^{-},a}\right),$$

$$\frac{d}{dt}N_{Ca^{2^{+}}}^{a}=\frac{1}{2F}\left(I_{NCX}^{Ca^{2^{+}},a}+I_{L}^{Ca^{2^{+}},a}\right),$$

$$\frac{d}{dt}N_{Glu}^{a}=\frac{1}{F}\left(I_{EAAT}^{Glu,a}+I_{L}^{Glu,a}\right),$$

$$\frac{d}{dt}W_{n}=L_{H_{2}O}^{n}RT\sum_{x}\left({\left[X\right]}_{n}-{\left[X\right]}_{e}\right),$$

$$\frac{d}{dt}W_{a}=L_{H_{2}O}^{a}RT\sum_{x}\left({\left[X\right]}_{a}-{\left[X\right]}_{e}\right).$$

We assume total volume and ion amounts to be constant for the entire simulation, by fixing constants *C*_X_ such that,


$$W_{e}=W_{tot}-W_{n}-W_{a},$$

$${\left[X\right]}_{e}=\left(C_{x}-N_{x}^{n}-N_{x}^{a}\right)/W_{e}$$

The simulations were performed at room temperature and used initial conditions (baseline resting conditions) and parameters as in Kalia et al. (2021). Under resting conditions, ion concentrations are stable, and all ion fluxes sum up to zero. This restriction was used to compute leak conductances for all ions that were otherwise not fixed.

### Experimental Design and Statistical Analysis

#### Fluorescence Intensity Decays (FID)

We used 3-mm-sized droplets of MQAE (3 mM) dissolved in an aqueous solution of 15, 40, or 100 mM chloride and imaged a 100-μm wide area located 50 μm inside the droplet. The photon number of FIDs are modified by changing either the acquisition time, excitation energy, or applied binning options. For binning, we summed the FIDs of pixels surrounding the central pixel FIDs, and assigned the FID sum as a new, binned FID to the central pixel (Figure 1).

#### MQAE Calibration

We repeated calibrations for all glial cell types in absence and presence of chemical blockers (bumetanide, DL-TBOA, or *R*-(+)-DIOA) (Kovalchuk and Garaschuk, 2012). The calibration curve is linear fitted to mean fluorescence lifetimes at five to eight preset chloride concentrations (10–60 mM; *N* = 3 animals/chloride concentration, >10 cells/mouse/chloride concentration). Error bars indicate the standard deviation (mean ± SD), in positive and negative direction. A linear fit of [Cl^–^] dependence of these values provides K_SV_ and τ_0_. The corresponding adjusted coefficient of determination ($R_{adj}^{2}$) of all linear fits was calculated as:


$$R_{adj}^{2}=1-\frac{\left(1-R^{2}\right)\cdot \left(n-1\right)}{n-k-1}$$

where *R*^2^ describes the percentage of the variation for a dependent variable that is explained by independent variables in a regression model, *n* is the number of observations and *k* is the number of independent variables. The $R_{adj}^{2}$ values are provided in Table 1 (Figures 2D,3B,4B,D,F,H).

**TABLE 1:** Calculated cell-type-specific K_SV_ (Stern-Volmer constants), τ_0_ (fluorescence lifetime in the absence of a quencher/chloride), and corresponding adjusted $R_{adj}^{2}$ (coefficient of determination) of all linear fits. MQAE calibration under physiological conditions and in the presence of bumetanide, DL-TBOA, or *R*-(+)-DIOA, results in substantial variation in characteristic K_*SV*_ parameters for MQAE, which reflects the chloride sensitivity of MQAE as well as in different τ_0_ values.

Brain Region	Parameter	physiological	Bumetanide (40 μM)	DL-TBOA (100 μm)	*R*-(+)-DIOA (100 μM)
CA1	K_SV_ in M^–1^	18.56	17.89	17.79	15.99
	τ_0_ in ns	6.63	5.42	5.73	5.51
	$R_{adj}^{2}$	0.991	0.963	0.996	0.993
DG	K_SV_ in M^–1^	16.85	22.06	17.35	17.69
	τ_0_ in ns	7.02	6.3	5.78	5.71
	$R_{adj}^{2}$	0.979	0.994	0.979	0.999
RGL	K_SV_ in M^–1^	20.66	22.65	18.65	17.42
	τ_0_ in ns	6.67	6.29	5.83	5.62
	$R_{adj}^{2}$	0.992	0.999	0.992	0.993
Cortex	K_SV_ in M^–1^	18.11	13.18	17.01	18.29
	τ_0_ in ns	6.24	5.07	5.49	5.54
	$R_{adj}^{2}$	0.976	0.983	0.991	0.989

*Abbreviations: CA1, cornu ammonis region 1; DG, dentate gyrus; RGL, radial glial-like cells.*

#### Chloride Concentration

**FIGURE 5:**
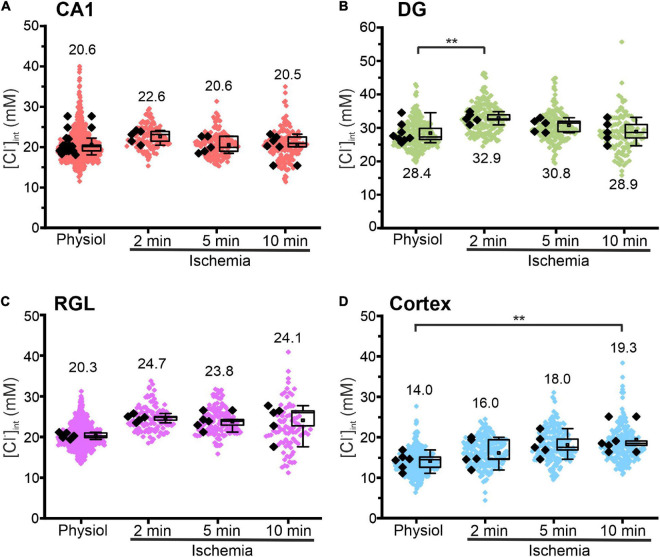
Modification of glial [Cl^−^]_int_ after energy restriction. [Cl^−^]_int_ of hippocampal astrocytes in the CA1 **(A)** and DG **(B)** regions, of RGL cells **(C)**, and of neocortical glial cells **(D)** under physiological conditions or after energy restriction for the indicated times. Black points are the mean [Cl^−^]_int_ from individual animals (mean ± SD) and colored points are the mean [Cl^−^]_int_ from individual cells. **p ≤ 0.01, one-way ANOVA with Holm–Sidak *post hoc* test; all *p*-values for this figure are mentioned in the results. Abbreviations: CA1, cornu ammonis region 1; DG, dentate gyrus; RGL, radial glial-like cells; Physiol, physiological; Ischemia, Chemical ischemia.

We calculated [Cl^–^]_int_ from τ_ave_ via calibration curves for individual cells after determining a ROI from individual glial cells in Fiji (Schindelin et al., 2012). In figures, box plots present data [mean ± 1.5 IQR (Interquartile Range)] as mean values from individual animals (black points) and single cells (small, shaded dots) without outlier exclusion (drawn in OriginPro 2018G; OriginLab Corporation, Northampton, United States). In the text, [Cl^–^]_int_ are given as the mean ± SD values from animals. For statistical analysis, one-way ANOVA tests with Holm–Sidak *post hoc* testing were used. Each experiment was repeated with at least four different animals (Figures 2E,F, 3C, 4A,C,E,G,5, 7, 8; numbers of mice, slices and cells are summarized in Supplementary Tables 1, 2, 4, 5).

#### Cell Volume Measurements Based on Maximum Intensity Projections

**FIGURE 6:**
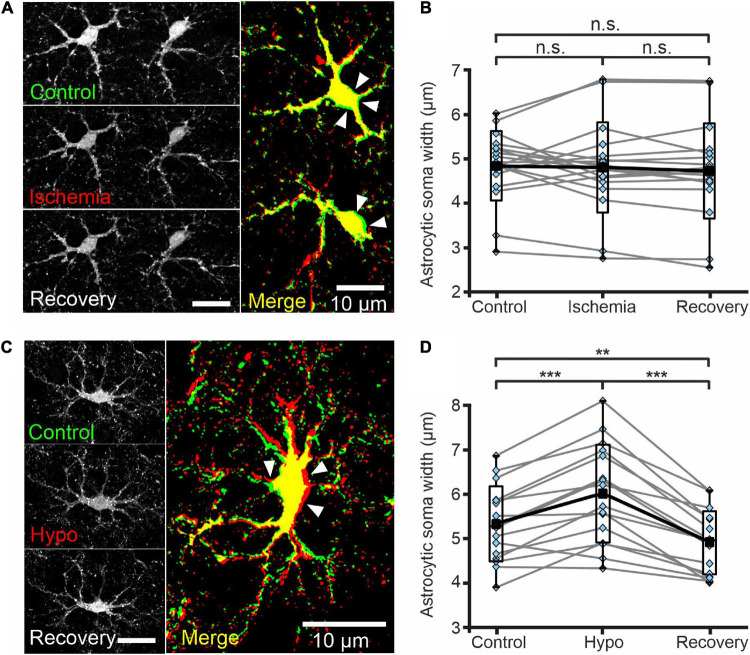
Astrocytes do not swell upon transient chemical ischemia. **(A)** Deconvolved confocal extended focus images showing SR101-labeled astrocytes in layer II/III of the mouse neocortex under control conditions, at 5 min after 10 min of chemical ischemia, and after 20 min of recovery. Arrowheads indicate areas of slight somatic shrinkage under chemical ischemia. The merged image is an overlay of the same cell under control (green) and ischemic (red) conditions **(B)** Box plots of astrocytic soma width (in μm) under the conditions described in panel **(A)**. **(C)** Deconvolved confocal extended focus images showing SR101-labeled astrocytes in layer II/III of the mouse neocortex under control conditions, at 5 min after a 10 min perfusion with hypo-osmotic saline (Hypo), and after 10 min of recovery (Recovery). The merged image is an overlay of the same cell under control (green) and hypo-osmotic (red) conditions. Arrowheads indicate areas of somatic swelling. **(D)** Box plots of astrocytic soma widths (as described in panel **(B)**) under the conditions described in panel **(C)**. Black squares are means, black lines are medians, whiskers cover min–max ranges, and boxes show the standard deviation; single data points are shown in blue. n.s. *p* ≥ 0.05, **p ≤ 0.01, ***p ≤ 0.001, one-way ANOVA with Bonferroni *post hoc* test; all *p*-values for this figure are mentioned in the results. Abbreviation: n.s.: not significant.

ImageJ (National Institutes of Health, Bethesda, MD, United States) and Origin Pro2018b (OriginLab 18 Corporation, Northampton, MA, United States) were used for image analysis and processing. Confocal z-stacks were sequentially taken at each experimental condition (control, treatment and recovery) to analyze astrocytic somata changes under hypo-osmotic and ischemic conditions. Based on earlier studies showing that astrocytic soma volume changes occur consistent in all directions (Risher et al., 2009; Vardjan et al., 2016), two-dimensional MIPs were generated. To this end, line profiles in scaled 8-bit MIP images were taken across somata to assess the changes in the diameter of individual astrocytes. Gray values were then normalized to the maximum, and the full width of half maximum (FWHM) of line plots was calculated. Absolute FWHM values were representative for the soma diameter and therefore indicative for cellular volume changes. Normality of datasets were tested using the Shapiro–Wilk test. One-way ANOVA of repeated measures with Bonferroni *post hoc* testing was used to compare data obtained in a single experimental series. Each experiment included at least five different animals (Figure 6; numbers of mice, slices and cells are summarized in Supplementary Table 3).

#### For All Experiments

All experiments were performed at room temperature (22–24°C). Our statistics are based on the comparison of individual animals as independent biological estimates. One might think that such statistical treatment might disguise small differences that could be observed by comparing individual cells before and after ATP restrictions. However, since glial cells are coupled to each other via gap junctions (Kettenmann and Ransom, 1988), they cannot be treated as independent biological estimates (Eisner, 2021). *p*-values of ≤0.05 were considered statistically significant with ^∗^*p* < 0.05, ^∗∗^*p* < 0.01, ^∗∗∗^*p* < 0.001. The exact *p*-values are provided in the results or relevant not significant results are marked with n.s. (not significant).

## Results

### Glial Cells Show Regional Variability in [Cl^–^]_int_

Figure 2 illustrates fluorescence lifetime imaging of acute MQAE-loaded hippocampal slices following two-photon excitation. CA1 and DG astrocytes and RGL cells were identified based on SR101 fluorescence (Figures 2A,B,C; Kafitz et al., 2008) and their characteristic morphology and localization. CA1 astrocytes were imaged in the stratum radiatum and DG astrocytes in the polymorphic layer at the interface between granule cell layers. The RGL subpopulation of glial cells are easily identified by SR101 staining owing to their characteristic unipolar morphology, with a single main extension projecting through the granule cell layer, and cell body located in the subgranular zone of the DG (Mori et al., 2006; Brunne et al., 2010; Jungblut et al., 2012). They express stem cell markers and can differentiate into either granule neurons or astrocytes (Berg et al., 2018).

MQAE fluorescence is collisionally quenched by Cl^–^ ions, resulting in an inverse linear relationship between fluorescence lifetime and chloride concentration (Verkman, 1990):


$$\frac{\tau_{0}}{\tau }\;=\;1+K_{SV}{\left[Cl^{-}\right]}_{int},$$

where τ is the MQAE fluorescence lifetime at a given [Cl^–^]_int_, τ_0_ is the MQAE fluorescence lifetime in the absence of chloride, and K_SV_ is the Stern–Volmer constant. MQAE is not only quenched by Cl^–^, so to account for the effects of other quenchers K_SV_ must be determined for each cell type. We calibrated MQAE lifetimes for various [Cl^–^]_int_ that were preset by permeabilizing the cell membrane with nigericin and tributyltin and then incubating cells in solutions containing 140 mM K^+^ and the chosen [Cl^–^] (Chao et al., 1989; Bevensee et al., 1997; Kaneko et al., 2004; Gensch et al., 2015; Untiet et al., 2017). Linear regression analysis of the average fluorescence lifetime at different intracellular chloride concentrations ([Cl^–^]_int_) derived the K_SV_ and τ_0_ for the three types of hippocampal glial cells (Figure 2D and Table 1). Using the obtained K_SV_ values, we calculated average [Cl^–^]_int_ values (Figure 2F) of 28.4 ± 3.0 mM in DG astrocytes, 20.6 ± 2.5 mM in CA1 astrocytes, and 20.3 ± 0.7 mM in RGL cells.

We next measured astrocytic [Cl^–^]_int_ in layer II/III of the neocortex (Figure 3). The K_SV_ for MQAE in neocortical astrocytes (Table 1) is in the same range as the K_SV_ values obtained for hippocampal CA1 astrocytes and RGL cells. However, the mean [Cl^–^]_int_ is much smaller in neocortical astrocytes (14.0 ± 2.0 mM) than in hippocampal astrocytes.

Taken together, our findings reveal a marked regional heterogeneity in resting [Cl^–^]_int_ in astrocytes. Moreover, average [Cl^–^]_int_ of hippocampal RGL cells is lower than that of cerebellar Bergmann glia, another class of radial glial cells (Untiet et al., 2017).

### Distinct Chloride Transport Processes Predominate in Hippocampal and Neocortical Astrocytes

We recently demonstrated that chloride accumulation via the NKCC1 electroneutral cation and chloride efflux through EAAT anion channels control [Cl^–^]_int_ of Bergmann glia cells (Untiet et al., 2017). EAATs are secondary-active glutamate transporters that can also function as anion channels (Fairman et al., 1995; Wadiche et al., 1995; Machtens et al., 2015; Fahlke et al., 2016). Glial cells additionally express the K^+^-Cl^–^ cotransporters KCC1 and KCC3 (Mount et al., 1999; Yan et al., 2001; Ringel and Plesnila, 2008) that mediate coupled potassium-chloride efflux. The Cl^–^/HCO_3_^–^ exchanger, AE3, is not expressed in glial cells (Kopito et al., 1989; Hentschke et al., 2006), and we therefore did not test whether blocking AE3 or Na^+^-HCO_3_^–^ transporters affect [Cl^–^]_int_.

We assessed the function of glial anion transporters in hippocampal and neocortical glial cells by measuring [Cl^–^]_int_ in the presence of specific inhibitors: i.e., 40 μM bumetanide (NKCC1 blocker) (Payne et al., 2003), 100 μM *R*-(+)-DIOA (KCC1–3 blocker) (Mercado et al., 2000), 20 μM UCPH-101 (EAAT1/GLAST blocker) (Abrahamsen et al., 2013), or 100 μM DL-TBOA (non-specific EAAT blocker) (Shimamoto et al., 1998; Figures 4A,C,E,G). This approach is complicated by blocker fluorescence and blocker quenching of MQAE fluorescence that modify MQAE lifetimes and prevent an accurate determination of [Cl^–^]_int_ from standard calibration results. Therefore, we performed MQAE calibration in the presence of bumetanide, DL-TBOA, or *R*-(+)-DIOA (Figures 4B,D,F,H), and observed substantial variation in characteristic Stern–Volmer parameters for MQAE (Table 1).

These experiments allow for distinction of two classes of glia cells that differ in responses to chloride transport blockers (Figures 4A,C,E,G). Whereas bumetanide decreased the [Cl^–^]_int_ to 10.9 ± 3.1 mM in CA1 (*p* = 7.91⋅10^–8^) and 19.9 ± 1.5 mM in DG (*p* = 5.51⋅10^–5^), it had no significant effect on the [Cl^–^]_int_ of neocortical astrocytes or RGL cells. On the other hand, *R*-(+)-DIOA increased [Cl^–^]_int_ in neocortical astrocytes to 26.6 ± 8.7 mM (*p* = 1.37⋅10^–4^) and RGL cells to 27.9 ± 4.2 mM (*p* = 1.15⋅10^–4^), but had no effect on the [Cl^–^]_int_ of CA1 or DG astrocytes.

Blocking EAAT1 by UCPH-101 increased [Cl^–^]_int_ most prominently in neocortical astrocytes (21.9 ± 2.3 mM; *p* = 0.008) and less effectively in CA1 astrocytes (25.0 ± 1.7 mM; *p* = 0.003) and RGL cells (24.4 ± 2.5 mM; *p* = 0.013), but had little effect on the [Cl^–^]_int_ of DG astrocytes. DL-TBOA blocks both glial glutamate transporters (EAAT1/GLAST and EAAT2/GLT-1) and increased [Cl^–^]_int_ to a greater extent than UCPH-101 in neocortical astrocytes (25.5 ± 4.1 mM; *p* = 3.37⋅10^–4^) and significantly increased the [Cl^–^]_int_ of RGL cells (32.8 ± 2.7 mM; *p* = 1.09⋅10^–8^). DL-TBOA had no apparent effect on astrocytic [Cl^–^]_int_ in the hippocampus.

We conclude that differences in the resting [Cl^–^]_int_ of different astroglial cell types are associated with differences in the number and/or activity of Cl^–^ import and export/efflux pathway proteins.

### Chemical Ischemia Has Only Slight Effects on Glial [Cl^–^]_int_

Na^+^-K^+^-ATPases are primary active transporters that generate Na^+^ and K^+^ gradients across glial cells. Since the majority of ATP is produced by oxidative phosphorylation, oxygen restriction reduces cellular ATP levels and Na^+^-K^+^-ATPase activity within short time. Reduced ATP may increase [Na^+^]_int_ and [K^+^]_out_ (Hertz et al., 2015; Gerkau et al., 2017) and diminish the membrane potential; it may thus modify all transport processes involved in the chloride homeostasis of glia cells. A major cause of energy restriction is brain ischemia, a neurological complication of vascular disease and a leading cause of disability and death in our aging population. The consequences of ischemia depend on severity of cell damage: extensive cell death occurs in the core ischemic zone, whereas cells in the ischemic penumbra may recover after reperfusion. We mimicked the effects of transient energy restriction in the ischemic penumbra by transiently removing glucose, blocking oxidative phosphorylation with sodium azide, and blocking glycolysis with 2-deoxy-D-glucose, followed by [Cl^–^]_int_ measurements under re-perfusion with oxygenated standard Ringer’s solution. In neocortical tissue slices, application of these components for 2 min induced intracellular ion disturbances (increase in Na^+^, Ca^2+^ oscillations) that were similar to those obtained during spreading depolarization waves in peri-infarct cortical regions in the rodent brain *in vivo* (Gerkau et al., 2018).

Figure 5 shows changes in the glial [Cl^–^]_int_ after chemical ischemia for 2, 5, and 10 min. Unexpectedly, in CA1 astrocytes and RGL cells, the [Cl^–^]_int_ remained constant at all three time points. In DG astrocytes, we observed a transient increase at 2 min (Figure 5B, *p* = 0.007) after chemical ischemia that returned to physiological levels at 5 or 10 min. Only in neocortical astrocytes, [Cl^–^]_int_ was increased after 10 min of chemical ischemia (Figure 5D, *p* = 0.008).

Cl^–^ is the main physiological anion and a major determinant of cell swelling and regulatory volume changes. Largely unaltered [Cl^–^]_int_ thus predicts the absence of significant volume changes during transient energy restriction. We therefore additionally measured astrocytic volumes in acute neocortical tissue slices stained with SR101 using MIPs from Z-stacks (0.6 μm increments) generated by confocal microscopy (Figures 6A,C). Chemical ischemia for up to 10 min (measured with 5 min delay) neither changed the mean width of astrocyte somata (Figure 6B, control-ischemia: *p* = 0.275; ischemia-recovery: *p* = 0.619; control-recovery: *p* = 0.894) nor caused strong delocalization or deformation of astrocytic extensions (Figure 6A). Notably, we observed increased soma sizes in some cells, but decreased sizes in others (Figure 6B). Since the absence of astrocytic volume changes during energy restrictions was surprising (Kimelberg, 2005), we transiently perfused slices with hypo-osmotic saline (220 mOsm/L) as control. Hypo-osmotic stress significantly increased the width of astrocyte somata from 5.3 ± 0.8 μm to 6.0 ± 1.1 μm (Figure 6D, control-hypo: *p* = 1.77⋅10^–5^; hypo-recovery: *p* = 2.54⋅10^–9^; control-recovery: *p* = 0.008), which was fully reversible (Figures 6C,D). In addition, primary and secondary astrocyte processes were strongly displaced (Figure 6C).

We conclude that glial [Cl^–^]_int_ and volumes undergo only slight changes under transient chemical ischemia of up to 10 min.

### Enhanced Chloride Accumulation and Chloride Efflux Can Compensate for Each Other Under Transient Energy Deprivation

**FIGURE 7:**
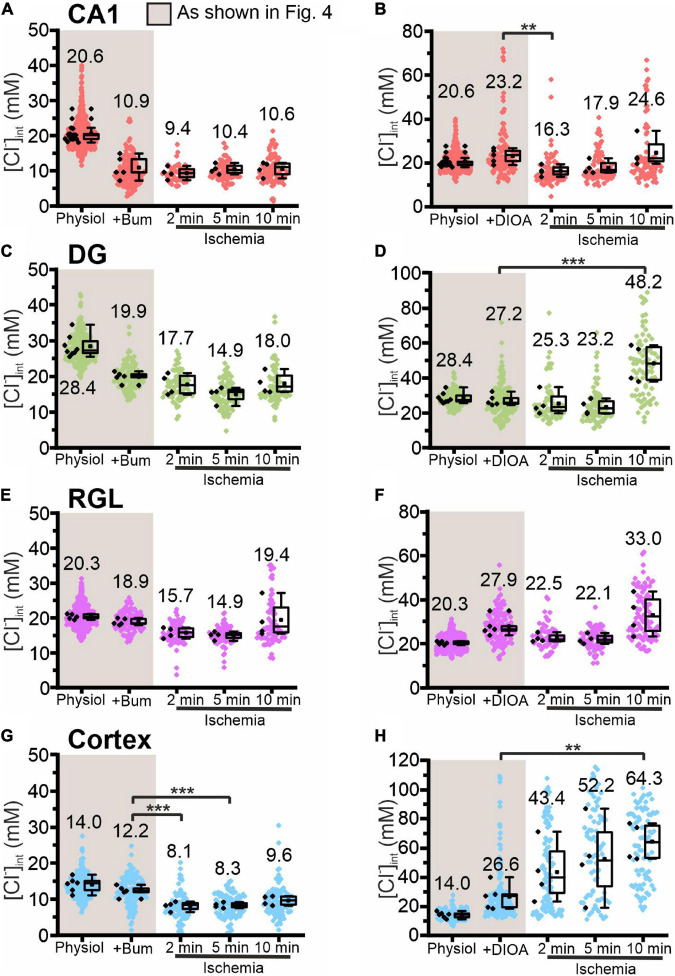
Modification of glial [Cl^−^]_int_ by a specific chloride transport blocker under energy restriction. [Cl^−^]_int_ of hippocampal astrocytes in the CA1 **(A,B)** and DG **(C,D)** regions, of RGL cells **(E,F)**, and of neocortical glial cells **(G,H)** under physiological conditions, in the presence of a specific chloride transport blocker (bumetanide or *R*-(+)-DIOA), or after energy restriction for the indicated times in the presence of the chloride transport blocker. In the box plot (mean ± 1.5 IQR), black points summarize the mean [Cl^−^]_int_ from individual animals and colored points represent the mean [Cl^−^]_int_ from individual cells. **p ≤ 0.01 and ***p ≤ 0.001, one-way ANOVA with Holm–Sidak *post hoc* test. Abbreviations: CA1, cornu ammonis region 1; DG, dentate gyrus; RGL, radial glial-like cells; Physiol, physiological; Ischemia, Chemical ischemia; Bum, bumetanide; DIOA, *R*-(+)-DIOA.

**FIGURE 8:**
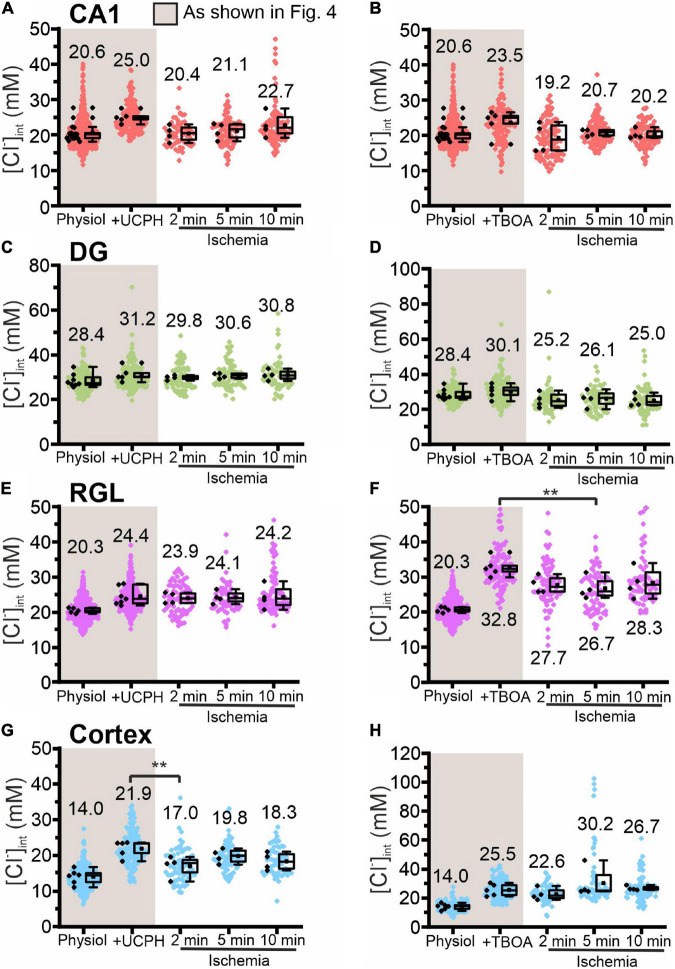
Modification of glial [Cl^−^]_int_ by a specific EAAT transporter blocker under energy restriction. [Cl^−^]_int_ of hippocampal astrocytes in the CA1 **(A,B)** and DG **(C,D)** regions, RGL cells **(E,F)**, and neocortical glial cells **(G,H)** under different conditions: physiological conditions, in the presence of a specific EAAT transporter blocker (UCPH-101 or DL-TBOA), and after energy restriction (ischemia) for the indicated times in the presence of the blocker. In the box plot (mean ± 1.5 IQR), black points are the mean [Cl^−^]_int_ from individual animals and colored points are the mean [Cl^−^]_int_ from individual cells. **p ≤ 0.01, one-way ANOVA with Holm–Sidak *post hoc* test; all *p*-values for this figure are mentioned in the results. Abbreviations: CA1, cornu ammonis region 1; DG, dentate gyrus; RGL, radial glia-like cells; UCPH, UCPH-101; TBOA, DL-TBOA.

To define the contribution of specific chloride transporters to glial [Cl^–^]_int_ during chemical ischemia, we measured the combined effect of restricted ATP supply and specific chloride transport blockers (Figures 7, 8). To achieve lasting inhibition of chloride transporters and channels, slices were continuously perfused with blocker-containing solutions (bumetanide, *R*-(+)-DIOA, DL-TBOA) during and after transient chemical ischemia. This was not necessary for UCPH-101 because of its slow unblocking kinetics (Abrahamsen et al., 2013).

Chemical ischemia had no pronounced effect on [Cl^–^]_int_ in CA1 and in DG astrocytes after pre-treatment with specific transport blockers (CA1: Figures 7A,B, 2 min: *p* = 0.005, Figures 8A,B; DG: Figures 7C,D, 8C,D). In DG astrocytes we observed an increase of the mean [Cl^–^]_int_ at 10 min after ischemic conditions and a pre-treatment with *R*-(+)-DIOA (Figure 7D, *p* = 1.94⋅10^–5^). In neocortical astrocytes, bumetanide alone had no effect under control conditions. After chemical ischemia, bumetanide decreased [Cl^–^]_int_ (Figure 7G, 2 min: *p* = 6.53⋅10^–4^, 5 min: *p* = 9.78⋅10^–4^), consistent with increased Cl^–^ efflux/outward transport pathways during energy restriction. *R*-(+)-DIOA, in contrast, caused a dramatic increase of [Cl^–^]_int_ under ischemic conditions (Figure 7H, 10 min: *p* = 0.002), but UCPH-101 (Figure 8G, 2 min: *p* = 0.006), but DL-TBOA did not (Figure 8H). Based on these results, we conclude that ischemia activates both NKCC1 and KCCs in neocortical astrocytes. Since these transporters mediate chloride transport in opposite directions, they partially compensate for each other, resulting in only a limited increase in the [Cl^–^]_int_ under energy restriction. Similar, but less pronounced effects were observed in RGL cells (Figures 7E,F and Figures 8E,F, 5 min: *p* = 0.005).

In these experiments we approximated glial cell volumes by defining a circular ROI surrounding the glial soma in FLIM images and assuming a spherical shape for the glial cell (Supplementary Figures 2, 3). This approximation did not reveal significant changes in volume under transient energy restriction. However, cell swelling was observed in neocortical astrocytes, when energy restriction in combination with selected anion transport blockers resulted in pronounced increases of [Cl^–^]_int_. Under similar conditions, we observed increased cell soma sizes, i.e., in the DG region after 10 min transient energy restriction combined with *R*-(+)-DIOA. Blocking EAAT1 and EAAT2 anion channels, respectively, lead only to slight volume changes in both directions. Cell swelling was not observed in other brain regions, probably due to only small changes in the [Cl^–^]_int_.

### Mathematical Modeling Reveals How Differences in Anion Transporter Numbers Can Cause Cell-Specific Responses to Transient Ischemia

To understand how changes in anion transporter activity shape the response to transient energy restriction, we used a recently developed quantitative model of a glutamatergic synapse to identify key determinants of synaptic failure during energy deprivation (Kalia et al., 2021). To account for the effects of *R*-(+)-DIOA-dependent KCC block on [Cl^–^]_int_, the model was expanded by inserting KCC cotransporters as an additional Cl^–^ transport system into astrocytes. Anion conductances generated by EAAT anion channels are represented as components of the glial leak anion conductance. Since the Cl^–^/HCO_3_^–^ exchanger, AE3, is not expressed in glial cells (Kopito et al., 1989; Hentschke et al., 2006), no HCO_3_^–^ transporter was integrated into the model. Neocortical and in DG astrocytes represent two extremes in both resting [Cl^–^]_int_ and changes in [Cl^–^]_int_ following the selective inhibition of anion transporters (Figures 2–5, 7, 8). As NKCC1 is the dominant Cl^–^ influx and KCC the dominant Cl^–^ efflux pathway in our model, we manipulated resting [Cl^–^]_int_ and changes of [Cl^–^]_int_ in response to energy restriction by varying the rates of these two transporters. With the exception of Cl^–^, Na^+^ and K^+^ leak conductances, all other parameters remained unchanged from the original model (Kalia et al., 2021).

Supplementary Figure 4 illustrates the iterative approach we used in optimizing these two parameters for the example of neocortical astrocytes. NKCC1 and KCC flux rates were varied around baseline values, which were either obtained from Kalia et al. (2021) for NKCC1 or set to $P_{\text{KCC}}^{a}=$1.3 ⋅ 10^–6^ $\frac{fmol}{msmV}$ for K^+^-Cl^–^ exchange. We modified normalized NKCC1 flux rates (*P^a*_NKCC_) between 1 and 100% and normalized KCC flux rates between 1 and 500% of these baseline values. For each set of *P^a*_KCC_ and *P^a*_NKCC_, changes in neocortical [Cl^–^]_int_ were calculated under resting conditions, as well as during and after transient energy restriction.

Variation of secondary active transporters requires adjustment of the leak conductances of the transported substrates to ensure that the sum of ion fluxes remains zero under resting conditions. Higher KCC and lower NKCC1 transport rates are thus associated with lower Cl^–^ leak conductances. At KCC rates exceeding the starting values, as well as for reduced KCC rates at NKCC1 rates below baseline, this limiting condition results in physiologically unreasonable negative values for astrocytic Cl^–^ conductances (Supplementary Figure 4A). For *P^a*_NKCC_ between 0.01 and 0.1 of the starting values, tenfold or hundredfold decreased KCC rates require implausibly low Cl^–^ leak conductances that result in continuously increasing [Cl^–^]_int_ after energy restriction (Supplementary Figure 4A). These predictions were in disagreement with experimental results, and these parameter values were thus discarded. The initial test thus demonstrates that *P^a*_NKCC_ must be around the starting values. The response of our model to energy restriction alone, however, did not suffice to restrain *P^a*_KCC_. We thus tested the effects of varying *P^a*_KCC_ at *P^a*_NKCC_ fixed to the baseline value on the consequences of pharmacological block of glial anion transporters (Supplementary Figure 4B). 3.5-fold increased *P^a*_KCC_ flux rates predicted best the effect of the KCC blocker DIOA on astrocytic [Cl^–^]_int_ in neocortical astrocytes and was thus used for further analyses.

**FIGURE 9:**
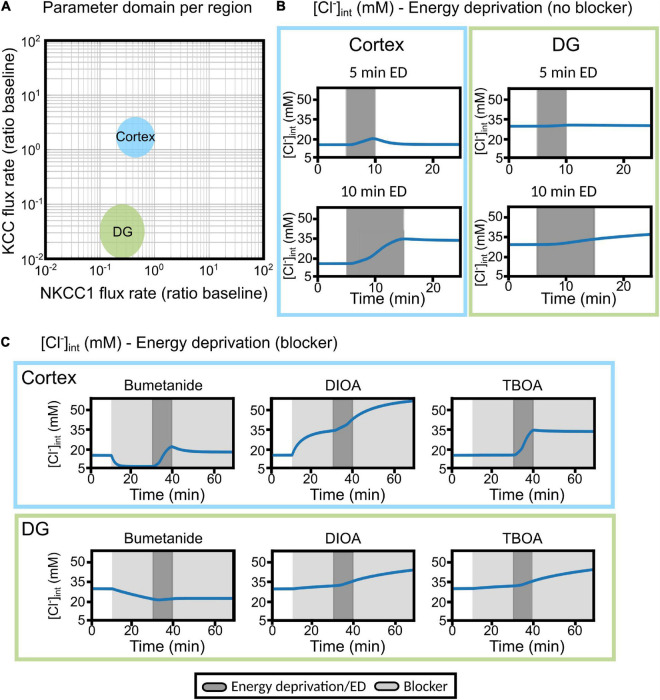
Differences in cortical and DG chloride homeostasis can be described by variation in glial KCC and NKCC1 expression levels in a mathematical model of the tripartite synapse. **(A)** Log–log plot of the KCC flux rate against the NKCC1 flux rate relative to baseline flux rates. Two distinct regions in this two-parameter space account for the results obtained with cortical (blue) or DG (green) [Cl^−^]_int_. The indicated regions correspond to parameter values with qualitative agreement with experimental traces. **(B)** Predicted changes in astrocytic [Cl^−^]_int_ without blocker under conditions used in experiments. Left panel, cortex; right panel; DG. **(C)** The model was subjected to block of a specific chloride transport system (light gray block: bumetanide, *R*-(+)-DIOA or DL-TBOA) for the first 30 min. At 20 min after the start of transport inhibition, transient ischemia (energy deprivation) was simulated by blocking neuronal and astrocyte Na^+^-K^+^-ATPase for 10 min (dark gray block), followed by energy restoration for 30 min in the presence of the mentioned blocker. Upper panel, cortex; lower panel, DG. Abbreviations: DG, dentate gyrus; ED, energy deprivation; DIOA, *R*-(+)-DIOA; TBOA, DL-TBOA.

Using this approach, we found two distinct parameter regions corresponding to [Cl^–^]_int_ measured in cortical and DG astrocytes (Figures 9B,C). They are shown in a log–log plot of the NKCC1 and KCC flux rates in Figure 9A: cortical results were well described by higher numbers in functional KCCs (approximately 10 times) in astrocytes than in neurons; and results for DG astrocytes were well described by lower numbers in both functional NKCC1 (about 10 times lower than in the cortex) and KCCs (about 100 times lower than neuronal KCC) (Figure 9A). Within the tested parameter ranges, no bifurcations were observed in the two-parameter space. Astrocytic [Cl^–^]_int_ smoothly changed upon parameter variation, and simulation results were consistent also for large perturbations of the parameters. The effect of DL-TBOA was simulated by blocking both neuronal and astrocyte glutamate transport and partially blocking astrocytic Cl^–^ leak currents (Figure 9C). In the cortex, DL-TBOA caused a sharp increase in [Cl^–^]_int_, followed by a plateau (at +200% of baseline). In contrast, in the DG DL-TBOA caused a slow rise to +5% of baseline at the end of the ischemic block. The EAAT anion conductance is modeled as part of a glial resting anion conductance (leak conductance). Since the exact contribution of EAAT anion channels to the total resting anion conductance is unknown, we varied the degree of resting conductance blockade by DL-TBOA, but found no discernible differences. These findings suggest that DL-TBOA mostly affects neuronal and glial glutamate transport, whereas changes in the glial resting conductance have only minor effects on [Cl^–^]_int_.

NKCCs and KCCs are both electroneutral transporters, so that only Na^+^ and K^+^ gradients across the membrane act as driving forces. Differences in NKCC and KCC flux rates may also be represented by variation in [Na^+^] and [K^+^], and one might thus be able to model the separate [Cl^–^]_int_ in DG and neocortical astrocytes by modifying cation transport rates. However, this possibility is excluded by experimental data. Intracellular [Na^+^] has been compared for both types of glia, with virtually identical results (Langer and Rose, 2009; Ziemens et al., 2019), and external [K^+^] does not vary under resting conditions. Thus, hippocampal and neocortical astrocytes are not expected to differ in cation transporter expression, and differences in [Cl^–^]_int_ are not due to separate driving forces for NKCCs and KCCs.

Moderate transient ischemia was initially simulated by blocking the Na^+^-K^+^-ATPase in both neurons and astrocytes to 50% of baseline activity (Kalia et al., 2021). In these simulations, astrocytes were subjected to energy deprivation for 5 or 10 min and then allowed to recover for 15 or 10 min, respectively (Figure 9B; gray shading). Additionally, astrocytes were treated with bumetanide (to block NKCC1), *R*-(+)-DIOA (to block astrocytic KCCs), or DL-TBOA (to block neuronal and astrocyte EAAT) for 20 min (light gray region), followed by energy deprivation for 10 min (dark gray block), and then another 30 min of transport block after energy restoration (Figure 9C). In response to transient ischemia for 5 min, [Cl^–^]_int_ in DG hippocampal astrocytes changed only slightly, whereas cortical astrocytes underwent a transient increase in [Cl^–^]_int_ that was fully reversible by restoring primary-active Na^+^-K^+^-transport after 5 min of energy restriction (Figure 9B). In response to 10 min energy restrictions, simulated [Cl^–^]_int_ rose to a larger extent than observed in experiments, for cortical as well as for DG astrocytes. However, experimentally observed differences between the two classes of astrocytes were reproduced; cortical astrocytes reacted with larger chloride accumulation to ATP restriction than DG astrocytes (Figures 5, 9B).

In DG astrocytes, bumetanide triggered Cl^–^ efflux under control conditions, and additional chemical ischemia changes the effects only slightly (Figure 9C, lower panel). *R*-(+)-DIOA caused small increases in [Cl^–^]_int_ after chemical ischemia in DG astrocytes. In cortical astrocytes, the model predicted a bumetanide-mediated reduction of [Cl^–^]_int_ that was partially reversed by energy restriction. This prediction differs from experimental observations, in which bumetanide had only minor effects under control conditions (Figure 4), but decreased [Cl^–^]_int_ in combination with chemical ischemia (Figure 7G). In simulations, *R*-(+)-DIOA caused massive Cl^–^ influx into the cortex (+300% of baseline), which was further enhanced by ischemic conditions and not reversed by energy restoration. This result is qualitatively, but not quantitatively similar to our experimental results (Figure 7H).

**FIGURE 10:**
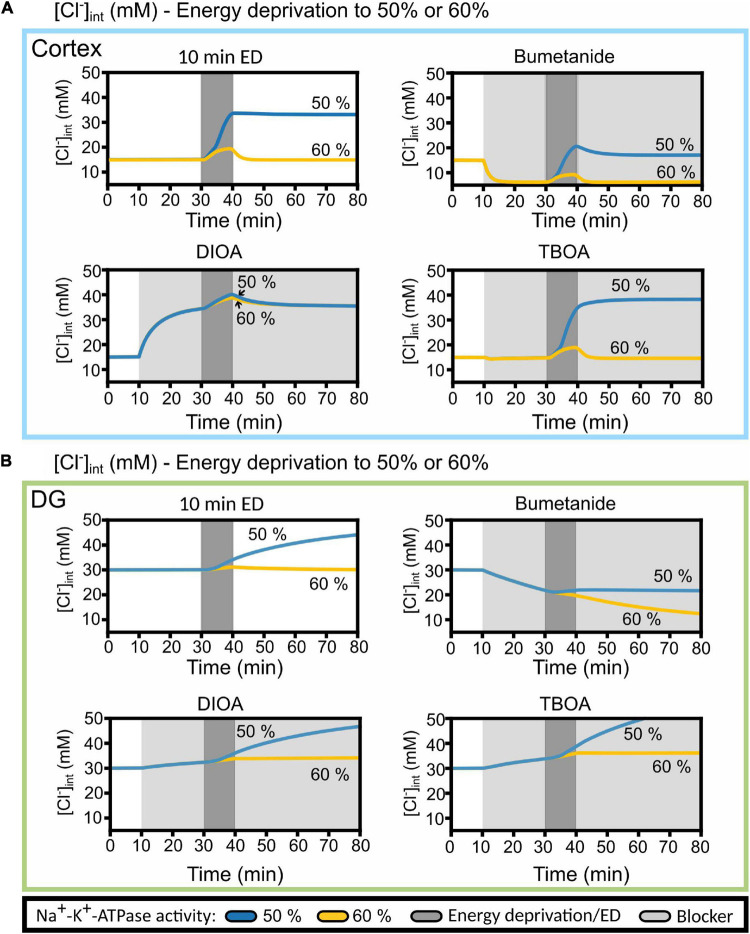
Predicted cortical and DG astrocyte chloride dynamics at Na^+^-K^+^-ATPase activity reduced to 50 or 60% of the baseline activity during chemical ischemia (energy deprivation). Time-dependent changes in [Cl^−^]_int_ for neocortical **(A)** and DG **(B)** astrocytes by reducing neuronal and astrocyte Na^+^-K^+^-ATPase activity for 10 min (dark gray block) to 50% (blue line) or to 60% (yellow line) of baseline. The model was subjected to block of a specific chloride transport system (light gray block: bumetanide, *R*-(+)-DIOA or DL-TBOA). At 20 min after the start of transport inhibition, transient ischemia was simulated followed by energy restoration for 40 min in the presence of the mentioned blocker. Abbreviations: DG, dentate gyrus; ED, energy deprivation; DIOA, *R*-(+)-DIOA; TBOA, DL-TBOA.

A possible explanation for the deviation between simulated and experimentally observed [Cl^–^]_int_ is provided in Figure 10, in which changes in [Cl^–^]_int_ are compared for blocking Na^+^-K^+^-ATPase to 60% baseline pumping capacity with the results upon 50% reduction from Figure 9. At higher remaining pump activity, the model predicts [Cl^–^]_int_ in DG astrocytes almost perfectly (Figure 10B). With less pronounced block of the Na^+^-K^+^-ATPase, the model predicts fully reversible changes in [Cl^–^]_int_ for cortical astrocytes after 10 min of chemical ischemia. In cortical astrocytes (Figure 10A), the combination of chemical ischemia for 10 min with bumetanide reduced [Cl^–^]_int_ to 6 mM in model astrocytes, closely similar to experimental results (Figure 7G). The effects of *R*-(+)-DIOA was comparable at 50 and 60% percentage remaining pump activity, and the TBOA effect was fully reversible at 60% rest activity.

Pronounced differences in [Cl^–^]_int_ changes upon small variations in Na^+^-K^+^-ATPase activity (Figure 10) are expected when simulating ion concentrations under ischemic conditions. Mathematical modeling demonstrated the existence of a tipping point of Na^+^-K^+^-ATPase pump activity that results in cell swelling and the establishment of a Gibbs–Donnan-like equilibrium state (Dijkstra et al., 2016; Kalia et al., 2021). After reaching this state, the re-establishment of the Na^+^-K^+^-ATPase does not bring the system back to its physiological state. Such a saddle-node bifurcation is characterized by large changes of the state variables, e.g., the ion concentrations, upon small variations of a model parameter close to the bifurcation point. It predicts that cells that differ only slightly in remaining ATP levels may exhibit pronounced differences in ion transport activities and in intracellular ion concentrations. Saddle-node bifurcation behavior predicts large inter-cell variability that makes mathematical modeling extremely complicated.

We conclude that our model qualitatively describes our experimental results under control conditions as well as under chemical ischemia. It shows that differences in the expression/activity of specific inward and outward chloride transporters can explain the experimentally observed region-specific differences in glial chloride homeostasis.

## Discussion

We used FLIM to study chloride homeostasis in hippocampal and neocortical glial cells in acute brain slices. We observed pronounced region- and cell type-heterogeneity in [Cl^–^]_int_ under both control conditions and chemical ischemia. The resting [Cl^–^]_int_ of astrocytes in the hippocampal CA1 (20.6 mM) was lower than in DG astrocytes (28.4 mM), but higher than in neocortical astrocytes (14.0 mM). The [Cl^–^]_int_ in RGL cells (20.3 mM) was comparable to values in CA1 astrocytes. These values are significantly lower than previously determined for cerebellar Bergmann glia cells with the same experimental approach (Untiet et al., 2017).

At present, we can only speculate about the physiological impact of the observed heterogeneity in glial [Cl^–^]_int_. Glial GAT transporters couple GABA uptake to Na^+^ and Cl^–^ gradients (Lu and Hilgemann, 1999). Lower astrocytic [Cl^–^]_int_ increases the driving force for GABA transport in the neocortex and may decrease synaptic and extrasynaptic [GABA] under certain conditions. Distinct glial [Cl^–^]_int_ might thus affect inhibitory synaptic transmission in these two brain regions. There is an increasing number of human diseases that are associated with altered intracellular chloride homeostasis (Uyanik et al., 2006; Kourdougli et al., 2017; Flores et al., 2019; Auer et al., 2020; Chivukula et al., 2020; Kovermann et al., 2020; Koumangoye et al., 2021). Our results suggest that changes in transporter function/expression will have different consequences in separate brain regions. Cation-chloride-cotransporters represent a possible target for the pharmacological treatment of such diseases (Kourdougli et al., 2017; Auer et al., 2020). Our experiments reveal that astrocytes in separate brain regions are distinctly sensitive to such blockers and that changes in glial [Cl^–^]_int_ might contribute to the therapeutic effects as well as to the side effects of such treatments. Lower [Cl^–^]_int_ may impair astrocytic volume regulation in the neocortex and may cause higher vulnerability of this brain region to ischemic damage than for example the hippocampus. However, this is not the case (Schmidt-Kastner, 2015), most likely since other factors such as the size of the extracellular space and expression levels of Na^+^-K^+^-ATPases play a more important role in defining vulnerability (Kalia et al., 2021).

We addressed mechanisms that determine baseline [Cl^–^]_int_ in the different types of glial cells using pharmacological inhibition (Figures 4, 7, 8) of chloride transporters as well as with mathematical modeling (Figures 9, 10). Blocking NKCC1 with bumetanide decreased the [Cl^–^]_int_ in hippocampal astrocytes, but not in neocortical astrocytes or RGL cells (Figures 4A,C,E,G). Whereas blockers of KCCs and EAAT chloride outward transport/efflux pathways did not affect the [Cl^–^]_int_ in DG astrocytes, K^+^-Cl^–^ co-transport as well as EAAT1/GLAST- and EAAT2/GLT-1-mediated anion currents were found to substantially contribute to [Cl^–^]_int_ in RGLs and neocortical astrocytes. In CA1 astrocytes, inhibition of EAAT anion channels, but not of KCCs, significantly changed the [Cl^–^]_int_. These results indicate that chloride accumulation by NKCC1, as well as chloride outward transport by KCCs and outward flux via two glial EAAT isoforms, EAAT1/GLAST and EAAT2/GLT-1, control glial [Cl^–^]_int_. The observed alterations in chloride concentration after blocking EAAT anion channels emphasize the importance of glutamate transporters for chloride homeostasis also for glial cells different from Bergmann glia (Untiet et al., 2017; Kovermann et al., 2020).

ATP shortage upon chemical blockade of oxidative and non-oxidative phosphorylation increases internal [Na^+^] (Gerkau et al., 2017, 2018) as well as external [K^+^] (Hertz et al., 2015) and [glutamate] (Belov Kirdajova et al., 2020). Altered [Na^+^] and [K^+^] modify driving forces for coupled transport by NKCC1 and KCCs, and raised [glutamate] increases the open probability of EAAT anion channels, thus affecting all major chloride transport pathways in glial cells and making changes in [Cl^–^]_int_ very likely. However, we did not observe pronounced changes in glial [Cl^–^]_int_ after transient chemical ischemia in our experiments (Figure 5). Absent [Cl^–^]_int_ changes are not due to a failure of our chemical ischemia protocol, since energy restriction affected internal chloride homeostasis in the presence of specific blockers (Figures 7, 8). Our data thus provide strong support for the notion that glial cells can counteract increased influx of chloride during transient energy restriction and thereby maintain [Cl^–^]_int_ unaffected in the first minutes. Since we have measured [Cl^–^]_int_ only in glial somata so far, we cannot predict how [Cl^–^]_int_ will vary in glial extensions under energy restrictions.

Transient energy restriction left glial [Cl^–^]_int_ and cell volume unaffected under a variety of conditions. Under all conditions that resulted in [Cl^–^]_int_ rise and cell swelling [but also under selected other conditions (Figure 6)], we observed high variability of these two values. Such variability is expected for ion homeostasis under energy restriction, where saddle-node bifurcation behavior predicts a threshold value for Na^+^-K^+^-ATPase activity, under which a pathological Gibbs-Donnan-like equilibrium state (Dijkstra et al., 2016; Kalia et al., 2021) develops, from which the system can only return at highly increased Na^+^-K^+^-ATPase rates. Under chemical conditions that decrease Na^+^-K^+^-ATPase activity to values close to this threshold value, there will be cells with pump activity below, and others with pump activity above this value. We thus expect cells with highly pathological and others with normal ion concentrations in our experiments, accounting for the observed variability of our experimental results under certain conditions.

We employed blocker experiments to understand how [Cl^–^]_int_ stays constant under energy restriction that modifies major chloride transport pathways. In neocortical astrocytes, the reduction in [Cl^–^]_int_ caused by bumetanide was greater under ischemic conditions (Figure 7G) than under control conditions in the same cells. In the presence of the KCC blocker *R*-(+)-DIOA (Figure 7H) ischemia caused the [Cl^–^]_int_ to increase. These results indicate that under ischemic conditions, KCC-mediated outward transport compensates for an increase in NKCC1-mediated inward transport. In DG astrocytes and RGL cells, chemical ischemia-induced stimulation of NKCC1 transporter does not result in major changes in [Cl^–^]_int_ in DG astrocytes and RGL after 2 and 5 min of energy restriction, however, it slightly increases chloride concentrations after 10 min (Figure 7D). Under all other experimental conditions, ischemia-mediated changes in [Cl^–^]_int_ in hippocampal astrocytes and RGL cells remained below experimental resolution limit. Blocking EAAT anion channels under ischemic conditions had only minor effects on [Cl^–^]_int_ (Figure 8), indicating that changes in EAAT anion current amplitudes are not major contributors to chloride homeostasis under ischemia.

At present, there is only a limited number of cation-chloride-cotransporter blockers available (Delpire, 2021), and the existing ones do not perfectly select between NKCC1 and KCCs. We used bumetanide at a concentration of 40 μM, and *R*-(+)-DIOA at 100 μM (Kelly and Rose, 2010). These concentrations are higher than those used for cultured cells [bumetanide: 0.5–5 μM (Delpire and Weaver, 2016); 10 μM; (Gillen and Forbush, 1999; Delpire, 2021); *R*-(+)-DIOA: IC_50_ ∼ 10 μM (Garay et al., 1988); 20 μM (Mercado et al., 2000; Shen et al., 2001)] simply to overcome the diffusion limits that slice preparations present. Bumetanide was reported to also inhibit KCCs, albeit with lower affinity (Gamba et al., 1994; Race et al., 1999; Mercado et al., 2000) than NKCC (Gillen et al., 1996; Gillen and Forbush, 1999; Hiki et al., 1999; Gamba and Bobadilla, 2004). We can therefore not fully exclude certain interactions of bumetanide and *R*-(+)-DIOA with other cation-chloride-cotransporters with opposite Cl^–^ transport direction. However, this does not invalidate our conclusion. Bumetanide decreases [Cl^–^]_int_ in CA1 and DG astrocytes, whereas *R*-(+)-DIOA is without effects (Figures 4A,C). This is only possible if reducing Cl^–^ inward transport is a limiting factor in setting [Cl^–^]_int_ in these cells. In neocortical astrocytes, *R*-(+)-DIOA increases [Cl^–^]_int_, and bumetanide is without effects (Figure 4G), indicating that KCC-mediated Cl^–^ outward transport is the limiting determinant of chloride homeostasis in these cells.

We routinely followed the volume of glial somata during our FLIM experiments and did not detect swelling under chemical ischemia. We verified these observations with MIPs by confocal microscopy (Figures 6A,C) and again neither observed changes in the width of astrocyte somata nor deformation of astrocytic extensions (Figure 6A) under these conditions. Since volume change require water fluxes based on electroneutral electrolyte fluxes, cell swelling is not possible without Cl^–^ fluxes and changes in [Cl^–^]_int_. The absence of major changes in [Cl^–^]_int_ (Figure 5) and in cell volume (Supplementary Figures 2, 3) upon energy restriction are thus fully consistent. However, when chemical ischemia is combined with certain anion transport blockers, [Cl^–^]_int_ and cell volume can increase. The most prominent example for such a change is the application of the KCC blocker *R*-(+)-DIOA to neocortical astrocytes (Figure 7H and Supplementary Figure 2H).

There exist marked differences in [Cl^–^]_int_ between the studied glial cells, ranging from 14 to 28 mM. The use of a mathematical model that describes ionic changes in the tripartite synapse under ischemia suggests a surprisingly simple cellular basis of the different responses of glial [Cl^–^]_int_ in cortex and hippocampus. We could reproduce the differences in resting [Cl^–^]_int_ as well as in ischemia and transport-block induced changes of this parameter by modifying numbers of functional NKCC1 and KCCs (Figure 9). Our results suggest that expression levels/activity levels of these two chloride transport systems are much higher in the cortex than in the hippocampus. Neocortical astrocytes exhibit lower [Cl^–^]_int_ than DG astrocytes despite higher levels of NKCC1, simply because of a larger number of functional KCCs. [Cl^–^]_int_ was assumed to be in dynamic equilibrium between Cl^–^ inward and outward movement. This simplifying view neglects the role of water co-transport through chloride channels and transporters and the importance of fixed charges inside and outside the cell in setting intracellular [Cl^–^] (Delpire and Staley, 2014; Glykys et al., 2014). However, although this simplification prevents exact quantification of relative numbers of cation-chloride-cotransporters in distinct glia types, it still permits predicting changes in [Cl^–^]_int_ upon block of distinct chloride transporters.

Na^+^-K^+^-2Cl^–^ cotransporter transport is regulated by external [K^+^] and internal [Na^+^] and [Cl^–^], as well as by cell shrinkage and by activation of metabotropic glutamate receptors (Delpire, 2000; Payne et al., 2003). Increased external [K^+^] and internal [Na^+^] are well established under experimental ischemic conditions. Since these changes affects K^+^ gradients more than Na^+^ gradients, they result in increased NKCC1 transport. The resulting changes in [Cl^–^]_int_ are counteracted by KCCs: after *R*-(+)-DIOA pre-treatment, we observed increased [Cl^–^]_int_ followed by cell swelling upon ischemia (Figure 7 and Supplementary Figure 2). Comparison of the experimental (Figures 4, 5, 7, 8) and modeling results (Figure 9) indicates that [Cl^–^]_int_ changes upon transient energy restriction can be fully described without assuming regulatory changes in the number of active transporters. Mere adjustment of individual NKCC1 and KCC transport rates by changing the driving force via altered ion concentrations predicts [Cl^–^]_int_ that are in good agreement with our experimental results. We assume that increased EAAT anion channel activity due to higher [glutamate] and changes in the membrane potential due to rises in [K^+^]_ext_ largely compensate for each other (Rossi et al., 2007; Rakers et al., 2017).

Since high water permeability permits rapid changes in glial cell volume upon osmotic variations (Andrew et al., 2007; MacAulay and Zeuthen, 2010; Nagelhus and Ottersen, 2013; Papadopoulos and Verkman, 2013), glial cells are generally assumed to be main drivers of brain swelling (Kimelberg, 2005). This notion is supported by the important role of TRPV4 activation for cerebral edema (Hoshi et al., 2018), and interactions between TRPV4 and the glial water channel aquaporin 4 (Benfenati et al., 2011). In neocortical slices at 32–34°C, Risher et al. (2009) observed astrocytic volume changes under oxygen/glucose deprivation for 10 min, which was rapidly reversible after re-oxygenation/normoglycemia. Neurons recovered much slower from swelling, and the authors assigned these differences between astrocytic and neuronal volume regulation to separate levels of aquaporin expression. In a subsequent *in vivo* study of brain ischemia, the same group (Risher et al., 2012) proposed that spreading depolarization can exacerbate astroglial swelling under energy restriction. Such consequences of neuronal dysfunctions were not addressed in our study. Benesova et al. (2009) distinguished two groups of astrocytes according to volume changes under oxygen/glucose deprivation. Both astrocyte groups exhibited an initial volume decrease of ∼10% during 20 min of oxygen/glucose deprivation, followed by only 5% volume increases in low response-astrocytes after reperfusion. Our simulation results illustrate how increases in [Cl^–^]_int_ and reversibility of ionic shifts depend on the duration of the energy restriction and the reduction in Na^+^/K^+^-ATPase activity (Figure 10). Neocortical astrocytes show slight increases in [Cl^–^]_int_ during energy restrictions that are fully reversible, whereas increased values do not reverse after 10 min. DG astrocytes only vary [Cl^–^]_int_ during 10 min of reduced Na^+^/K^+^-ATPase function (Figure 9).

Secondary-active transporters exhibit temperature-dependent transport rates (Dalmark and Wieth, 1972; Lacko et al., 1973; Schäfer and Heber, 1977), and glial [Cl^–^]_int_ is expected to change with temperature, since intracellular chloride concentrations are established as dynamic equilibrium of different transport processes. For highly regulated transport proteins such as the NKCCs and the KCCs as main determinants of intraglial chloride homeostasis, not only transport rates, but also regulatory processes are affected by temperature. For the KCCs, a reduction in transport rates upon higher temperatures was reported in heterologous expressions systems (Hartmann and Nothwang, 2011), likely because of such temperature-dependent regulatory processes (Jennings and al-Rohil, 1990; Hannemann and Flatman, 2011). These features prevent extrapolating the chloride concentrations at physiological temperatures from our results. However, quantifying ion concentrations with fluorescent indicators in acute brain slices at 37°C – especially under blocking conditions – is extremely difficult. We observed excessive cell swelling in preliminary, physiological experiments at higher temperatures that made accurate FLIM measurements of [Cl^–^]_int_, which require scanning times of at least 40 or 80 s, impossible. In fact, the vast majority of studies on acute slices were performed at room temperature (Rungta et al., 2015; Jennings et al., 2017; Langer et al., 2017; Gerkau et al., 2019; Breithausen et al., 2020; Lerchundi et al., 2020) or at 31–32°C (Glykys et al., 2014; Kolbaev et al., 2020).

Our study focuses on understanding mechanisms of chloride homeostasis in glial cells, making blocking experiments and mathematical modeling a central part of our analysis. We modeled glial [Cl^–^]_int_ using a recently developed biophysical model (Kalia et al., 2021) that describes ion dynamics in neurons and astrocytes at glutamatergic synapses and was developed based on ion concentrations experimentally determined at room temperature. Moreover, a recent study comparing Na^+^ signals in acute slices under chemical ischemia and *in vivo* imaging in peri-infarct cortex an *in vivo* model demonstrated that the chemical ischemia protocol used in our study at room temperature nicely resembles results of ischemic penumbra regions in living animals (Gerkau et al., 2018). We therefore chose to use the non-physiological room temperature that permitted accurate quantification of FLIM results under all tested conditions and made conditions, in which chloride transport pathways are partially blocked, amenable to our experiments.

In summary, we have quantified intracellular [Cl^–^] in various glia cell types under resting conditions and transient energy restriction. None of the tested cell types underwent major changes in [Cl^–^]_int_ during transient chemical ischemia. Thus, glia cells can control internal chloride concentrations and support cell volume stability under transient energy deprivation.

## Data Availability Statement

The raw data supporting the conclusions of this article will be made available by the authors, without undue reservation.

## Ethics Statement

The animal study was reviewed and approved by German Law for Protection of Animals and European Community Council Directive 2010/63/EU and were approved by the regulatory authorities, the FZJ/HHU and Landesamt für Natur, Umwelt und Verbraucherschutz of Nordrhein-Westfalen, and Central Unit for Animal Research and Animal Welfare Affairs of the Heinrich Heine University Düsseldorf, in accordance with institutional act number O50/05.

## Author Contributions

ME, MK, HM, MP, TG, CR, and CF conceived the project. ME, CR, and CF wrote the manuscript with input from all other authors. ME, MK, SR, and LP performed the research and analyzed the data. PK supported mouse work and statistical analysis. All authors contributed to the article and approved the submitted version.

## Conflict of Interest

The authors declare that the research was conducted in the absence of any commercial or financial relationships that could be construed as a potential conflict of interest.

## Publisher’s Note

All claims expressed in this article are solely those of the authors and do not necessarily represent those of their affiliated organizations, or those of the publisher, the editors and the reviewers. Any product that may be evaluated in this article, or claim that may be made by its manufacturer, is not guaranteed or endorsed by the publisher.
