# Supplementary material for: Glial Chloride Homeostasis Under Transient Ischemic Stress
Source: Front Cell Neurosci. 2021 Sep 16;15:735300. doi: 10.3389/fncel.2021.735300 (PMC8481871; doi:10.3389/fncel.2021.735300)
Supplement: Supplementary file 1 [file Data_Sheet_1.pdf]

Supplementary Figures and Tables

1.1 Glial cell volumes

Approximated glial cell volumes by defining a circular region of interest (ROI) surrounding the glial soma in FLIM (fluorescence lifetime imaging microscopy) images and assuming a spherical shape for the glial cell soma.

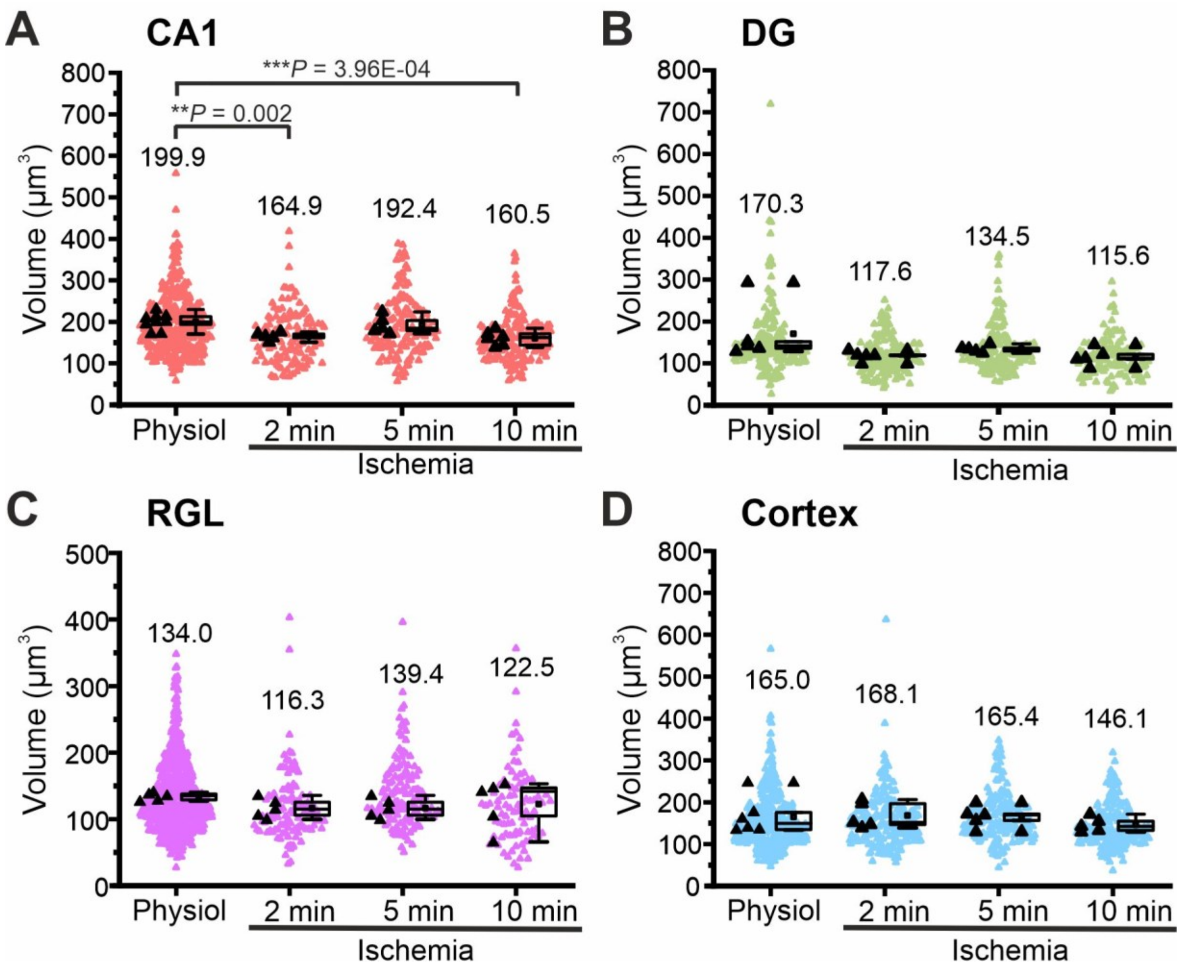

Supplementary Figure 1. Moderate energy restriction does not result in major changes in volume. Mean glial soma volumes (μm^3) of hippocampal astrocytes in the CA1 (**A**) and DG (**B**) regions, of RGL cells (**C**), and of neocortical glial cells (**D**) under physiological conditions or after energy restriction (ischemia) for the indicated times (2, 5 or 10 min). Glial somata sizes were calculated by defining circular ROIs surrounding the glial somata in FLIM images. Black triangles summarize the mean volumes from individual animals (mean ± SD) and colored triangles represent the mean volumes from individual cells. $**p \leq 0.01$ and $***p \leq 0.001$; one-way ANOVA with Holm–Sidak *post hoc* test. Abbreviations: CA1 – cornu ammonis region 1; DG – dentate gyrus; RGL – radial glial-like cells; Physiol: physiological; Ischemia: Chemical ischemia.

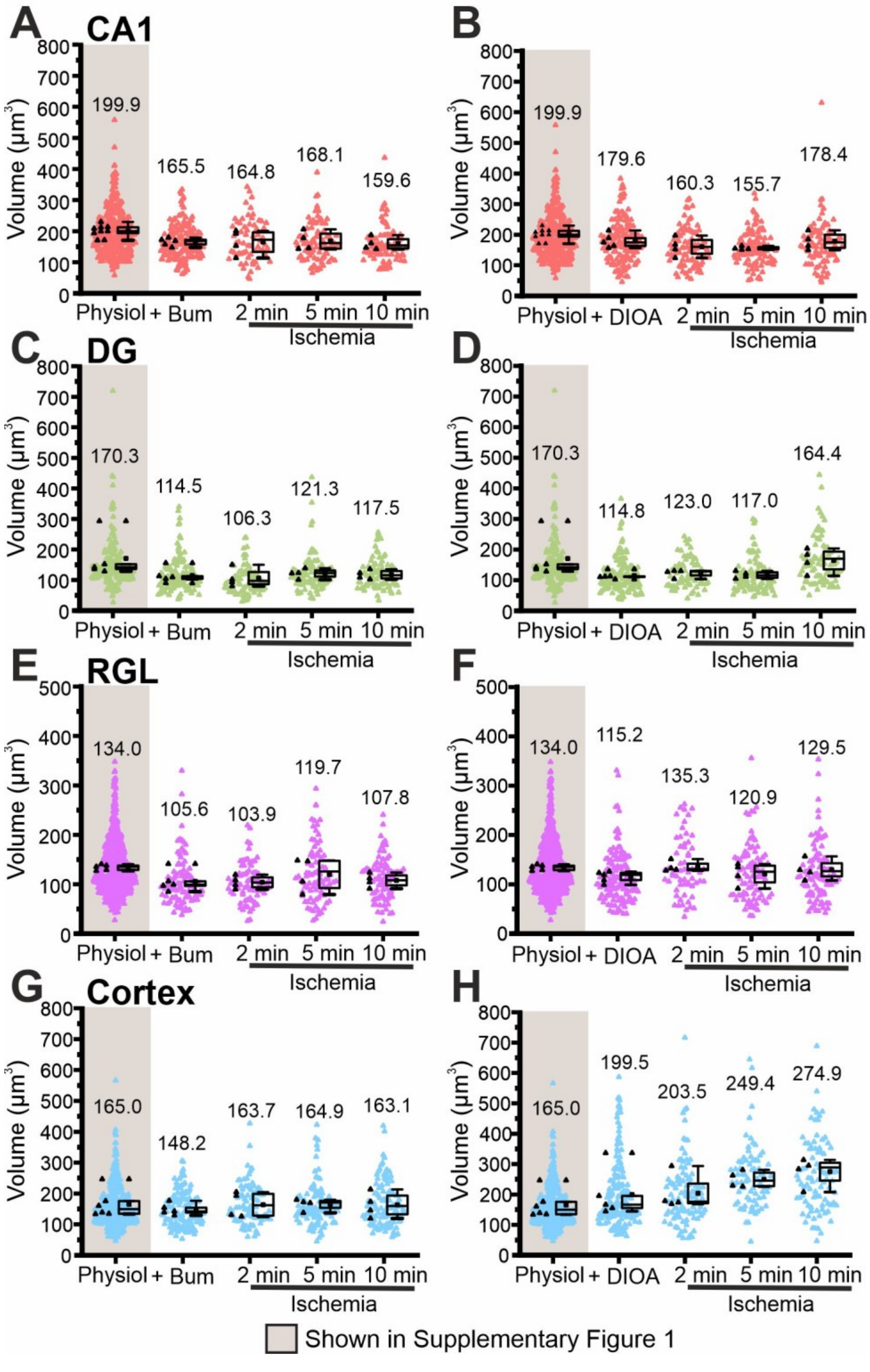

Supplementary Figure 2. Moderate energy restriction does not result in significant changes in glial soma sizes in the presence of anion transport blockers – bumetanide or R- (+)-DIOA. Mean glial soma volumes (μm^3) of hippocampal astrocytes in the CA1 (**A,B**) and DG (**C,D**) regions, of RGL cells (**E,F**), and of neocortical glial cells (**G,H**) after energy restriction (ischemia) for the indicated times (2, 5, or 10 min) and presence of the indicated blockers. Glial somata sizes were calculated by defining circular ROIs surrounding the glial somata in FLIM images. Black triangles summarize the mean volumes from individual animals (mean ± SD) and colored triangles represent the mean volumes from

individual cells. Abbreviations: CA1 – cornu ammonis region 1; DG – dentate gyrus; RGL – radial glial-like cells; Physiol: physiological; Ischemia: Chemical ischemia; Bum: bumetanide; DIOA: R- (+)-DIOA.

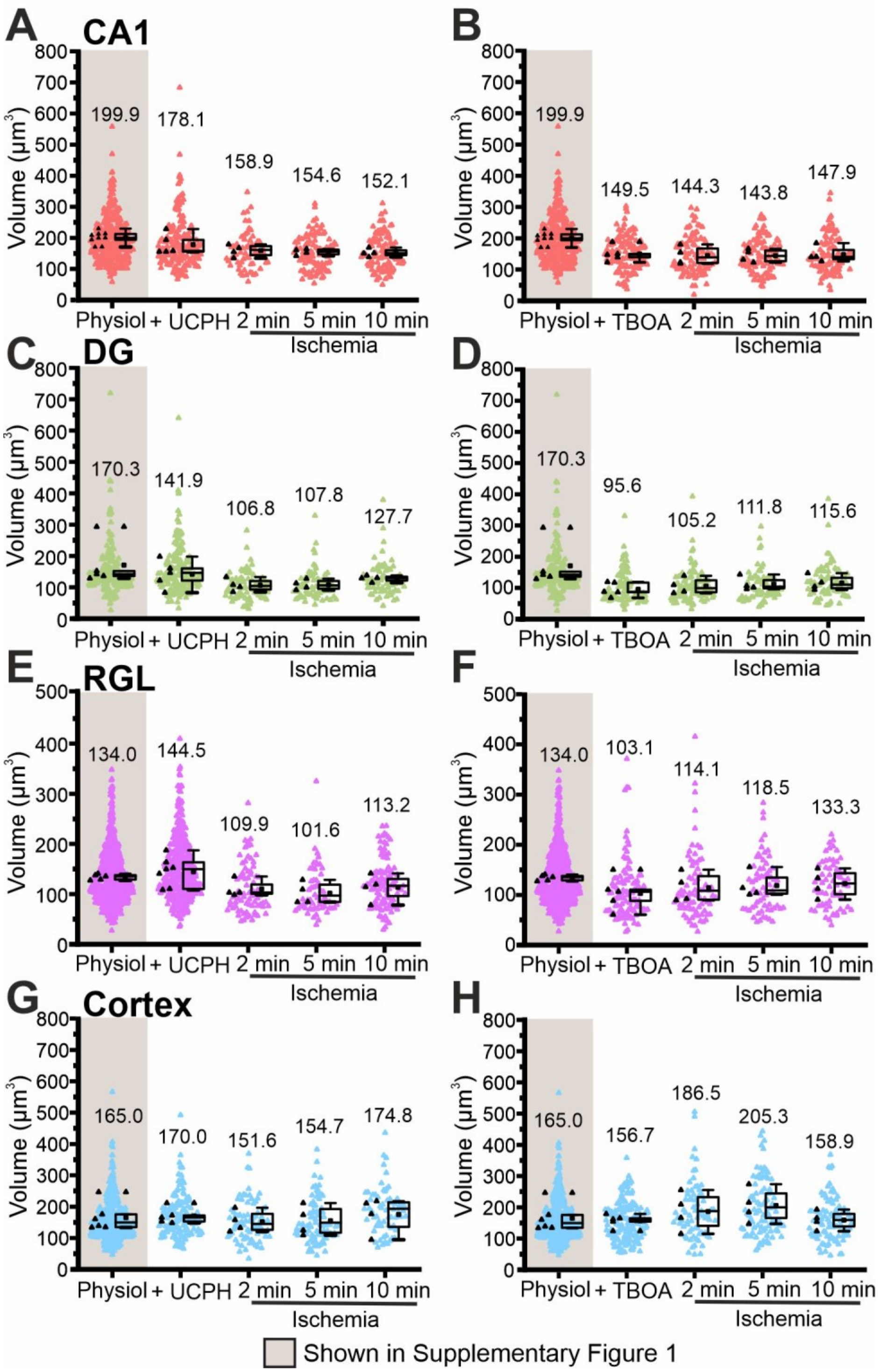

Supplementary Figure 3. Moderate energy restriction does not result in significant changes in glial soma sizes by blocking excitatory amino acid transporter/s – UCPH-101 or DL-TBOA. Mean glial soma volumes (μm^3) of hippocampal astrocytes in the CA1 (**A,B**) and DG (**C,D**) regions, of RGL cells (**E,F**), and of neocortical glial cells (**G,H**) after energy restriction (ischemia) for the indicated times

(2, 5, or 10 min) and presence of the indicated blockers. Glial somata sizes were calculated by defining circular ROIs surrounding the glial somata in FLIM images and calculating the volumes by assuming the glial cells as spherical. Black triangles summarize the mean volumes from individual animals (mean ± SD) and colored triangles depict the mean volumes from individual cells. Abbreviations: CA1 – cornu ammonis region 1; DG – dentate gyrus; RGL – radial glial-like cells; Physiol: physiological; Ischemia: Chemical ischemia; UCPH: UCPH-101; TBOA: DL-TBOA.

1.2 Modeling $[Cl^-]_{int}$ under transient ischemia

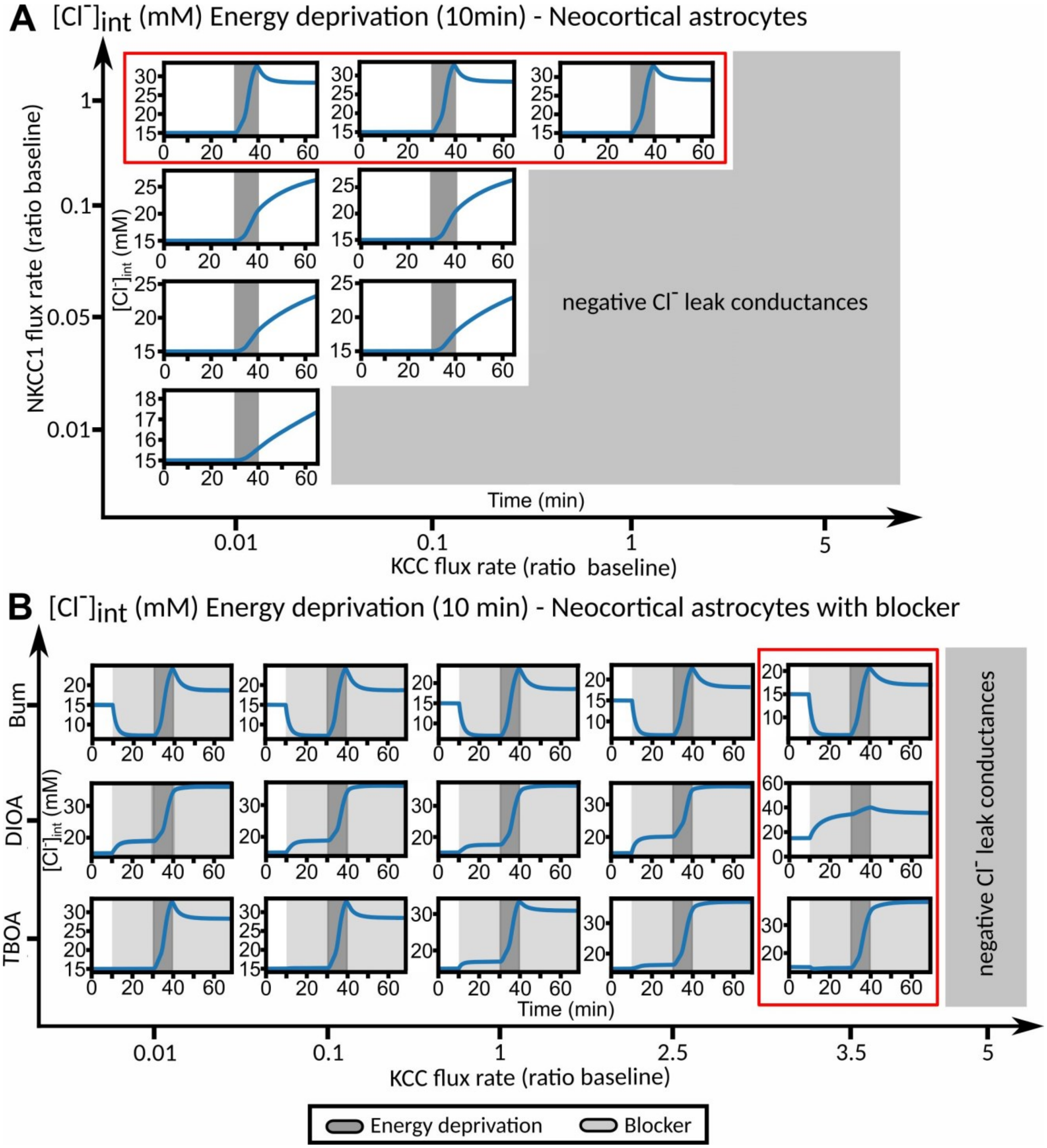

Supplementary Figure 4. Iterative optimization of NKCC1 and KCC flux rates in neocortical astrocytes. (**A**) Predicted $[Cl^-]_{int}$ under resting conditions and changes during or after energy restriction for various normalized KCC and NKCC1 flux rates. NKCC1 flux rates (P^a_{NKCC}) were varied between 1% and 100% of baseline values and the KCC flux rate (P^a_{KCC}) between 1% and 500% of baseline values. Changing KCC and NKCC1 rates requires re-calibration of leak conductances to ensure stable resting $[Cl^-]_{int}$. In some cases, this procedure results in physiologically unreasonable negative chloride leak conductances. Only NKCC1 rates close to the baseline value correctly predict the experimentally observed changes in $[Cl^-]_{int}$ upon energy restriction. (**B**) Predicted changes in astrocytic $[Cl^-]_{int}$ under resting conditions, under blocking conditions (light gray block: bumetanide, *R*-(+)-DIOA or DL-TBOA) as well as during or after energy restriction for multiple KCC normalized flux rates (0.01-5) at fixed baseline P^a_{NKCC}. At 20 min after the start of transport inhibition, transient ischemia (energy deprivation) was simulated by blocking neuronal and astrocyte Na^+-K^+-ATPase for 10 min (dark gray block), followed by energy restoration for 30 min in the presence of the mentioned blocker. For a KCC flux rate that is 3.5 times the baseline value, the predictions of the model are in best agreement with experimental results with blockers as well as with blockers under energy restriction. Red boxes show in (**A**) and (**B**) evaluated flux rates for neocortical astrocytes. Abbreviations: Bum: bumetanide; DIOA: R- (+)-DIOA; TBOA: DL-TBOA.

1.3 N-numbers (mice, slices, cells)

Abbreviations: CA1 – cornu ammonis region 1; DG – dentate gyrus; RGL – radial glial-like cells; Physiol: physiological; Ischemia: Chemical ischemia; Bum: bumetanide; DIOA: R- (+)-DIOA; UCPH: UCPH-101; TBOA: DL-TBOA

Supplementary Table 1. N-numbers of glial resting $[Cl^-]_{int}$ (Figure 2E,F; Figure 3C) and in the presence of the indicated blockers (Figure 4 A,C,E,G).

	N-numbers (mice, slices, cells)			
	CA1	DG	RGL	Cortex
Physiol	17/42/529	8/18/222	5/24/597	6/19/365
Bum	5/14/155	5/13/122	5/13/94	5/11/123
DIOA	5/12/142	5/14/139	5/14/132	5/14/185
UCPH	5/15/174	5/14/175	7/29/393	5/13/141
TBOA	5/10/120	5/12/116	5/12/105	5/13/163

Supplementary Table 2. N-numbers of glial resting $[Cl^-]_{int}$ and after energy restriction (Figure 5).

	N-numbers (mice, slices, cells)			
	CA1	DG	RGL	Cortex
Physiol	17/42/529	8/18/222	5/24/597	6/19/365
2 min Ischemia	5/10/124	5/14/169	5/14/108	5/14/208
5 min Ischemia	5/11/150	5/14/169	5/14/83	5/16/197
10 min Ischemia	6/13/167	5/11/121	5/11/86	5/17/198

Supplementary Table 3. N-numbers of cell volume measurements based on maximum intensity projections (Figure 6).

Cortex	N-numbers (mice, slices, cells)		
6 B		6 D	
Ischemia	5/5/18	Hypo-osmotic stress	7/13/16

Supplementary Table 4. N-numbers of glial resting $[Cl^-]_{int}$, in the presence of the indicated blockers and under energy restriction (Figure 7).

	N-numbers (mice, slices, cells)			
	CA1	DG	RGL	Cortex
Physiol	17/42/529	8/18/222	5/24/597	6/19/365
Bum	5/14/155	5/13/122	5/13/94	5/11/123
+ 2 min Ischemia	4/5/63	4/6/76	4/6/76	4/5/85
+ 5 min Ischemia	4/6/81	4/8/90	4/8/81	4/7/102
+ 10 min Ischemia	4/6/86	4/6/76	4/6/80	4/7/106
	N-numbers (mice, slices, cells)			
	CA1	DG	RGL	Cortex
Physiol	17/42/529	8/18/222	5/24/597	6/19/365
DIOA	5/12/142	5/14/139	5/14/132	5/14/185
+ 2 min Ischemia	4/7/87	4/6/67	4/6/61	4/7/100
+ 5 min Ischemia	4/7/101	4/7/93	4/7/86	4/6/77
+ 10 min Ischemia	4/7/89	4/7/76	4/7/90	4/7/86

Supplementary Table 5. N-numbers of glial resting $[Cl^-]_{int}$, in the presence of the indicated blockers and under energy restriction (Figure 8).

	N-numbers (mice, slices, cells)			
	CA1	DG	RGL	Cortex
Physiol	17/42/529	8/18/222	5/24/597	6/19/365
UCPH	5/15/174	5/14/175	7/29/393	5/13/141
+ 2 min Ischemia	4/6/64	4/7/81	4/7/74	4/6/73
+ 5 min Ischemia	4/7/99	4/7/75	4/7/58	4/8/88
+ 10 min Ischemia	4/7/98	4/7/72	4/7/100	4/6/63
	N-numbers (mice, slices, cells)			
	CA1	DG	RGL	Cortex
Physiol	17/42/529	8/18/222	5/24/597	6/19/365
TBOA	5/10/120	5/12/116	5/12/105	5/13/163
+ 2 min Ischemia	4/8/97	4/8/98	4/8/77	4/7/73
+ 5 min Ischemia	4/8/98	4/7/66	4/7/66	4/7/89
+ 10 min Ischemia	4/8/95	4/7/69	4/7/64	4/7/91
